# First-in-Human Trial of a Novel Suprachoroidal Retinal Prosthesis

**DOI:** 10.1371/journal.pone.0115239

**Published:** 2014-12-18

**Authors:** Lauren N. Ayton, Peter J. Blamey, Robyn H. Guymer, Chi D. Luu, David A. X. Nayagam, Nicholas C. Sinclair, Mohit N. Shivdasani, Jonathan Yeoh, Mark F. McCombe, Robert J. Briggs, Nicholas L. Opie, Joel Villalobos, Peter N. Dimitrov, Mary Varsamidis, Matthew A. Petoe, Chris D. McCarthy, Janine G. Walker, Nick Barnes, Anthony N. Burkitt, Chris E. Williams, Robert K. Shepherd, Penelope J. Allen, for the Bionic Vision Australia Research Consortium

**Affiliations:** 1 Centre for Eye Research Australia, University of Melbourne, Royal Victorian Eye and Ear Hospital, East Melbourne, Australia; 2 Bionics Institute, East Melbourne, Australia; 3 Department of Medical Bionics, University of Melbourne, East Melbourne, Australia; 4 Department of Pathology, University of Melbourne, St Vincent's Hospital Melbourne, Fitzroy, Australia; 5 NICTA, Computer Vision Research Group, Canberra, Australia; 6 National Institute for Mental Health Research, Australian National University, Canberra, Australia; 7 Centre for Neural Engineering, University of Melbourne, National Information and Communications Technology Australia (NICTA), Ltd., Melbourne, Australia; Saitama Medical University, Japan

## Abstract

Retinal visual prostheses (“bionic eyes”) have the potential to restore vision to blind or profoundly vision-impaired patients. The medical bionic technology used to design, manufacture and implant such prostheses is still in its relative infancy, with various technologies and surgical approaches being evaluated. We hypothesised that a suprachoroidal implant location (between the sclera and choroid of the eye) would provide significant surgical and safety benefits for patients, allowing them to maintain preoperative residual vision as well as gaining prosthetic vision input from the device. This report details the first-in-human Phase 1 trial to investigate the use of retinal implants in the suprachoroidal space in three human subjects with end-stage retinitis pigmentosa. The success of the suprachoroidal surgical approach and its associated safety benefits, coupled with twelve-month post-operative efficacy data, holds promise for the field of vision restoration.

**Trial Registration:**

Clinicaltrials.gov NCT01603576

## Introduction

Blindness is one of the most feared and debilitating physical disabilities [1], with the World Health Organisation estimating that 285 million people worldwide have vision impairment and 39 million people are blind [2]. Recent technological advances have allowed the development of visual prostheses, or “bionic eyes”, which use implanted electrodes within the visual pathway to restore rudimentary vision to those with severe vision loss [3], [4]. The success of these technologies has been such that two devices are now commercially available, with European CE mark approval being granted to the Argus II device (Second Sight Medical Products, USA) in 2011 followed by FDA approval in early 2013, and the Alpha IMS device (Retina Implant AG, Germany) gaining CE mark approval in mid-2013. These advances have provided proof of concept for the field of vision restoration, and brought hope to millions of people who have lost their sight from disease or trauma.

Visual prostheses work by using implanted electrodes to stimulate neurons in the visual pathway with electrical current via an implanted stimulator [5], [6] or independent microphotodiode-amplifier-electrode elements [7], [8]. The majority of visual prostheses developed to date use electrodes implanted within or near the retina to directly stimulate the inner retinal neurons, bypassing the non-functioning or dead photoreceptors, and utilizing the intact posterior visual pathway to transmit signals to the visual cortex. Thus, retinal prostheses require a relatively intact inner retinal layer to be beneficial, such as seen in retinal degenerative diseases like retinitis pigmentosa (RP) [9]. RP accounts for over 1.5 million cases of blindness and is the most common form of inherited blindness [10]. RP causes degeneration of vision over varying time periods and primarily targets the photoreceptor cells in the outer retina, with anatomical and retinal imaging studies showing that the inner retinal neurons are relatively preserved in these people [9], [11]–[13].

**Figure 1:**
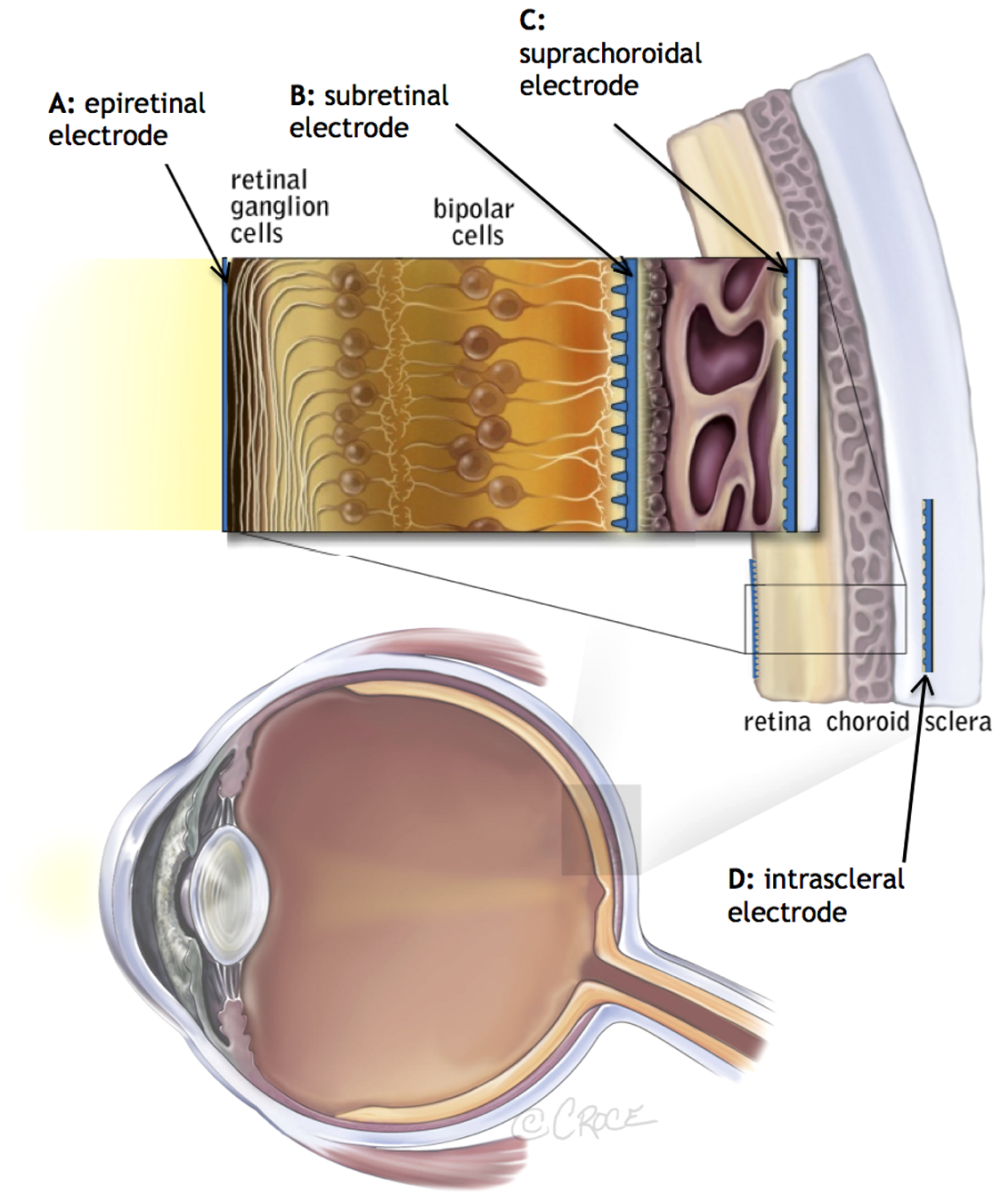
Potential anatomical locations for retinal prosthesis implantation. To date, clinical trials have been performed with devices in the A: epiretinal position [15], B: subretinal space [7] and D: intrascleral space [19]. Image modified with permission from Bionic Vision Australia.

Previous work has shown that retinal prostheses in the epiretinal, subretinal and intra-scleral locations (Fig. 1) can provide patients with perceptions of light and basic object recognition, with some recipients even able to read large print [7], [8], [14]–[19]. However, there have been some challenges with previous approaches including issues with stability of the implant, conjunctival and scleral erosions, retinal detachment, hypotony and endophthalmitis [15], [20], [21]. Many of these complications are related to the device location and required implantation procedures, which include vitrectomy (removal of the gel-like vitreous humour from within the eye) and highly technical surgical techniques. Such adverse events can lead to poor vision outcomes, loss of residual vision or the need for revision/removal surgery.

The aim of this study was to develop a less invasive, more anatomically stable implantation position, by placing the array between the firm fibrous sclera and the outer retina/choroid, in the suprachoroidal space (Fig. 1C).

One of the main benefits with this location is that the surgical placement is less technically challenging and does not breach the retinal tissue, negating the need for a vitrectomy or incisions into the retina itself. The main disadvantage with this location is that the electrodes are 250 to 400 µm further away from the target retinal ganglion cells than in an epiretinal implant [22]. However, our group has shown in a preclinical feline model that not only is the suprachoroidal surgical approach feasible, reproducible and safe [23], the prosthesis is biocompatible, mechanically stable [24] and the electrode array is efficacious, with the ability to produce cortical responses within acceptable stimulation safety parameters [25], [26]. There have also been human studies showing that intra-scleral electrodes, which are implanted even further from the ganglion cells, are able to safely evoke phosphenes in patients with end-stage retinal degeneration at levels of stimulation considered to be safe for platinum electrodes [16].

This report describes the first human phase 1 clinical trial (clinicaltrials.gov, NCT01603576), involving three participants implanted with a retinal prosthesis in the suprachoroidal space. This study was designed as a proof of concept, first-in-human trial of the suprachoroidal implant. To allow for maximum flexibility in device stimulation, a percutaneous connector was used to allow direct stimulation of the intraocular electrode array, with no implantable electronics used. The percutaneous connector has previously been used in cochlear implant studies [27] and requires similar surgical procedures as the implantation of a bone-anchored hearing aid. Full specifications of the device are detailed in the following Methods section.

## Materials and Methods

The study protocol and supporting TREND checklist are available as supporting information; see S1 Protocol and S1 TREND Checklist.

### Patient screening and selection

After approval from the Human Ethics Committee of the Royal Victorian Eye & Ear Hospital (application # 11/1032H) and trial registration (www.clinicaltrials.gov, trial # NCT01603576), three subjects aged 49, 52 and 63 years were enrolled in the study. Subjects were identified through a screening process at the Centre for Eye Research Australia, where 95 people with RP participated in a series of research projects as part of Bionic Vision Australia's development of the bionic eye.

An extensive battery of screening tests was used to assess suitability for the study, including visual performance tests (visual acuity, movement discrimination, Goldmann visual fields), confirmation of diagnosis through medical reports and clinical examination, measurement of residual retinal function using advanced Fourier transform analysis of electroretinography [28], assessment of the retina and choroidal structure using optical coherence tomography [22], assessment of functional vision in the home environment by an orientation and mobility instructor and an optometrist, psychosocial screening tests (including assessments of quality of life [29]) and a comprehensive consultation with a rehabilitation psychologist.

Initially, patient informed consent was provided for an 18-month study, but due to the successful progress in the first year (reported in this paper) and the availability of new hardware to enhance patient testing, a 6-month extension was offered to all participants at the twelve-month mark. All participants reconsented to the extension, making the entire study two years in duration. The second half of the study, including results of mobility testing and device explantation, will be detailed in a follow-up report. Independent psychological support was provided at all stages of the study and, in particular, during the informed consent periods.

The inclusion/exclusion criteria for this study are outlined in Table 1.

**Table 1:** Inclusion/exclusion criteria.

Inclusion Criteria	Exclusion Criteria
Aged 18 years or older	Any co-existent ocular disease, with the exception of mild cataracts
Either gender	Inability to visualize the retina due to corneal or other ocular media opacities (corneal degenerations, dense cataracts, trauma, lid malposition)
A confirmed history of outer retinal degenerative disease such as retinitis pigmentosa or choroideremia	Any ocular condition that predisposes the subject to rubbing their eyes
Remaining visual acuity of bare light perception or less in both eyes	Cognitive deficiencies, including dementia or progressive neurological disease
Functional inner retina (ganglion cells and optic nerve), as shown by the ability to perceive light and/or a measurable corneal electrically evoked visual response	Psychiatric disorders, including depression, as diagnosed by a qualified psychologist
A history of at least 10 years of useful form vision in the worse seeing eye	Deafness or significant hearing loss, or the presence of a cochlear implant
Must be willing and able to comply with the testing and follow-up protocol demands (preferably residing within 1.5 hours of the investigational site)	Poor general health or pregnancy, which would exclude them from obtaining a general anesthetic

The three selected subjects provided written consent after being provided with both audio and electronic versions of the consent information. The subjects were a 52 year old post-menopausal female with rod-cone dystrophy and approximately 20 years of light perception vision (P1), a 49 year old male with syndromic RP (Bardet-Beidl syndrome) and approximately 8 to 10 years of light perception vision (P2), and a 63 year old male with rod-cone dystrophy and approximately 20 years of light perception vision (P3). All subjects were guide dog users. P2 and P3 had significant nystagmus, which did affect the ability to obtain high quality ocular images. P3 also had controlled epilepsy, for which he was taking valproate sodium. He was closely monitored by a neurologist during the study, and there was no evidence that the surgery or device stimulation changed his condition in any way.

All three subjects were assessed on four separate occasions over a two-year period prior to recruitment in the study whilst participating in research studies examining the natural progression of retinitis pigmentosa. The repeated measures allowed us to ensure that their baseline level of vision was stable, as people with RP often experience fluctuations in their visual performance [30], [31], and it is imperative to obtain an accurate baseline assessment of visual function prior to any vision restoration treatment.

### The suprachoroidal retinal prosthesis implant

**Figure 2:**
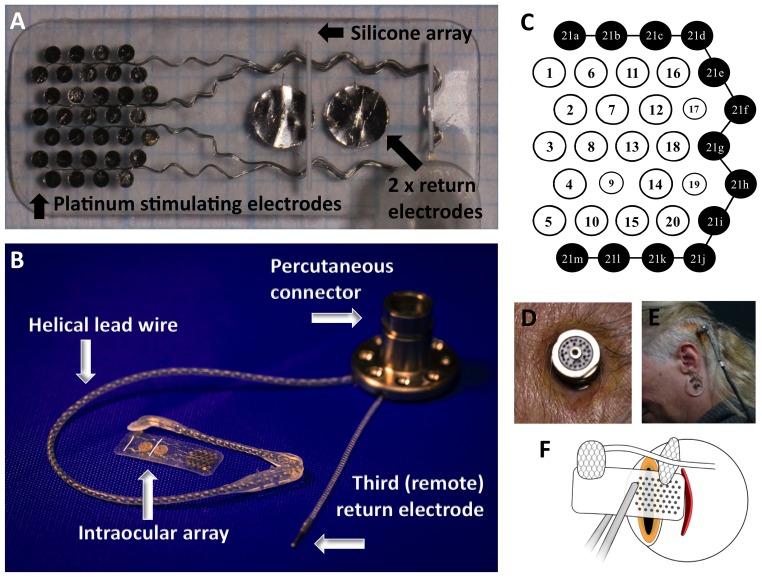
The intraocular electrode array of the suprachoroidal device (A) and the entire device (B), showing the array connected to the percutaneous connector via a helical lead wire. The electrodes on the intraocular array (C) were numbered for analysis, with the black electrodes (21a to 21m) being ganged to provide an external ring for common ground and hexagonal stimulation parameter testing. Note electrodes 9, 17 and 19 were smaller (400 µm vs. 600 µm). The percutaneous connector protruded through the skin behind the ear (D), allowing direct connection to the neurostimulator via a connecting lead (E). The scleral incision was made 9 mm to 10 mm posteriorly from the sclero-corneal limbus.

The device used in this study consisted of an intraocular electrode array (Fig. 2a), composed of a silicone substrate with 33 platinum stimulating electrodes (30×600 µm diameter, 3×400 µm diameter) and 2 large return electrodes (2000 µm diameter). The electrodes were recessed 50 µm from the silicone surface. A third return electrode was implanted remotely subcutaneously behind the ear. The outer ring of stimulating electrodes was ganged together to allow investigation of the use of hexagonal stimulation [32], meaning that there were a total of 20 electrodes that could be individually stimulated (Fig. 2c). The array was 19 mm long and 8 mm wide.

The intraocular array was connected by a helical lead wire to a titanium percutaneous connector (Fig. 2b), which was implanted behind the subject's ear. The lead wire was 150 mm long and contained 23 individually insulated platinum/iridium wires. The 24^th^ wire from the remote platinum return connected directly to the percutaneous connector.

The percutaneous connector has previously been used in cochlear implant studies [27] and required similar surgical procedures as the implantation of a bone-anchored hearing aid. The connector was anchored to the skull using titanium screws, and protruded through the skin behind the ear (Fig. 2d). The advantage with the percutaneous connector was that it enabled direct stimulation of the electrode array via a connecting lead (Fig. 2e), without the need for implantable electronics. This allowed unparalleled flexibility in the testing paradigms possible.

### Suprachoroidal surgical technique

The surgical procedures for the implantation of the suprachoroidal retinal implant and the percutaneous connector were developed as an iterative process, incorporating both cadaver studies (feline and human) and acute and chronic (3 months) implantation in preclinical studies using a feline model [33]. An acute study was also completed in a human patient who was undergoing enucleation due to an unrelated ocular condition. This acute study enabled surgeons to trial the procedure with an inactive mock device prior to the commencement of the clinical trial. Full details of the surgical procedures can be found in a previous publication by Saunders et al [33], and are summarized here.

All three operations occurred at the Royal Victorian Eye and Ear Hospital, Victoria, Australia between May and August 2012, with vitreoretinal surgeons PA, JY and MM and ENT surgeon RB. General anaesthesia was administered and the area was prepared near the site of the percutaneous connector by shaving the hair and sterilizing the location with Betadine. The eye was also prepared with a Betadine wash. A dummy electrode array and percutaneous pedestal were used at this early stage of surgery to plan and mark the required incisions.

A curved scalp incision was used to expose the posterior temporalis muscle, followed by an incision through the muscle to expose a flat section of squamous temporal bone. The periosteum was dissected from the bony site to allow attachment of the percutaneous pedestal. A tunnel was created beneath the temporalis fascia between the percutaneous connector location and the lateral orbital rim.

A lateral canthotomy was performed and extended towards the ear and an incision made in the flap for the percutaneous portion of the device. After the lateral orbital margin was exposed, the periosteum was incised and a flap created. A lateral orbitotomy was created with 10 mm burrs below the zygomaticofrontal suture for lead stabilization. The electrode array was loaded into a purpose built trochar and then passed forward to the lateral canthotomy/lateral orbital margin. The percutaneous pedestal was secured with self-tapping screws and the external ground electrode was placed beneath the temporalis periosteum close to the pedestal.

The scleral incision was made behind the lateral rectus muscle, to use the muscle to cover the Dacron patch and reduce the risk of exposure. As such, a temporal peritomy was performed and the lateral rectus muscle identified. Lateral rectus was disinserted and tied with 6/0 vicryl (Ethicon). The scleral wound position was marked with diathermy (as decided from preoperative axial length measurements, ultrasonography and magnetic resonance imaging). The location of the scleral wound is demonstrated in Fig. 2f. Due to the axial length, globe dimensions and lateral rectus muscle insertion location, P3 had a more posterior incision location (10 mm posterior to the sclera-corneal limbus) than P1 and P2 (both 9 mm posteriorly from the limbus). This meant that the electrode array was placed more temporal from the fovea for P3 than the other subjects. A full thickness scleral wound was made with a 15 degree blade (Alcon) and the suprachoroidal space initially dissected with a crescent blade (Alcon), then with a lens glide (bvi Visitec 581001).

The electrode array was inserted into suprachoroidal space and the superior wound edge was extended posteriorly in an L shape to allow the lead to exit and the Dacron patch to sit flat on the globe. This L shaped extension allowed the device position to be optimized during insertion. The wound was closed with 8/0 nylon (Ethicon) and the Dacron patch sutured to the sclera with 8/0 nylon.

After the scleral wound was closed, the lateral rectus muscle was reattached. The device position was assessed with indirect ophthalmoscopy. Due to the subretinal hemorrhage noted three days postoperatively in P1, we attempted to minimize eye movements in P2 and P3 in the immediate post-operative recovery period by injecting Botulinum toxin Type A (Allergan Australia, NSW, Australia) into the four extraocular rectus muscles. This did not change the outcome for P2 and P3, with both also experiencing a mild to moderate subretinal bleed in the 3–4 day postoperative window.

At the conclusion of the surgery, the conjunctiva was closed with 8/0 vicryl. The lead was fixated in an orbitotomy drilled in the frontal process of the zygomatic bone, and held in place by a specifically designed silicone grommet. The periosteum was closed over this with 6/0 vicryl sutures. The subcutaneous tissue wound was closed with 6/0 vicryl and the skin sutured with 5/0 nylon or staples.

An injection of subtenon's local anaesthesia was used for short-term pain relief and chloramphenicol antibiotic ointment was applied to the eye. Finally, a pressure dressing was applied to the area surrounding the percutaneous connector. This was removed the following day.

Subjects remained in hospital for four to five days of postoperative care before being discharged. They were given postoperative topical steroid (predneferin forte) and antibiotic (chloramphenicol) eye drops, and analgesia as required. After discharge, subjects were reviewed weekly and monitored for adverse events, post-operative safety and clinical efficacy as described below.

### Assessment of surgical safety and efficacy

Intraoperative safety was defined as the number of adverse events during the surgical procedure. Surgical efficacy was defined as the proximity of the electrode array to the fovea following implantation, and the mechanical stability of the device in the suprachoroidal space. Both of these assessments were made from quantitative measurements on ocular imaging modalities (fundus photographs and optical coherence tomography).

### Assessment of post-operative safety

Post-operative safety was assessed by the number of serious device related adverse events following the implantation. We defined a serious adverse event as one that required altered or increased medical management, such as surgery or hospitalization. To be considered as “device-related” there needed to be a direct or indirect causal link between the device, implantation surgery or study protocols and the adverse outcome.

### Assessment of device and clinical efficacy

Device efficacy was determined by the number of electrodes that remained connected and viable after implantation (as indicated by electrode impedances) and the ability for subjects to perceive repeatable phosphenes when the device was stimulated.

Electrode impedance was measured at the end of the cathodic (first) phase of a biphasic current pulse and defined as the quotient of dividing the peak measured voltage by the supplied current [34]. Individual electrode impedances were measured using clinical software running on a laptop PC. Measurements were made intraoperatively to ensure that the implant was functional before closing the surgical wounds. After surgical recovery, impedance measurements were made using the same laptop-based system approximately fortnightly for the duration of the study.

Visual function was assessed via two tests – the Basic Assessment of Light and Motion (BaLM) test [35] and the Landolt C optotype recognition subtest from the Freiburg Acuity and Contrast Test (FrACT) [36], both of which were presented to subjects in a darkened room (108–114 lux) using a 30-inch computer monitor placed at 57 cm viewing distance. The BaLM test was completed with all subjects, but only P1 completed the Landolt C testing protocol in the initial 12-month post-operative period. Testing incorporated a head-mounted video camera with a manufacturer stated field-of-view of 67°×50.25° (Arrington Research Inc., Scottsdale AZ, USA) and a pixel dimension of 320 by 240 pixels. Within the implant, the 20 stimulating electrodes are arranged in a staggered grid measuring 3.5 mm×3.46 mm, corresponding to a visual field projection on the retina of 12.4°×12.2° [37].

The BaLM test involves detection of a light wedge in one of four quadrants, and assesses the ability of the device to improve light localisation skills [35]. Given the response options were four alternative forced choices (4AFC), the chance rate was 25% and the criterion cutoff for success set at 62.5%. The BaLM was completed with the device off (control) and device on. The device on setting used a vision processing algorithm called Lanczos2 filtering, which is reported in detail elsewhere [38], [39]. In short, the Lanczos2 filter is a reconstruction filter that is used in downsampling and translating the high resolution image from the camera to electrode stimulation parameters. The filter ensures artefacts from such downsampling do not appear, such as a flickering which may result from making small head movements with the camera viewing fine detail. This makes objects appear more consistent in their appearance. The percentage of accurate responses in the BaLM test was determined for each participant with device on and device off. Comparisons between participants and device setting for percentage of accurate responses were calculated using χ^2^ statistics. Binomial distributions were calculated to determine whether the accuracy rates were significantly better than chance (i.e., 25%). Windows SPSS v22 (IBM Corporation, Somers, NY) was used for all statistical analyses.

The Landolt C optotype recognition test used was a subtest from the FrACT [36], which is freely available online (http://michaelbach.de/fract/index.html). This task requires subjects to identify the orientation of a letter C, and can be used to estimate visual acuity. It was not possible to present appropriately sized optotypes on our 30-inch computer monitor, so a region-of-interest was defined within the camera video image to be 18×18 pixels, corresponding to a reduced visual field of 3.77°×3.77° (a ‘zoom’ of 3.26). Measured thresholds have this correction factor applied. The theoretical acuity for this device was assumed to be the minimum grating distance that can be represented in four orientations. This is limited by the 1 mm horizontal electrode spacing, and corresponds to a grating of 0.141 cycles-per-degree, or 2.33 logMAR (20/4242). We only completed the Landolt C test on P1, and she was tested over 32 sessions. In total, this testing involved 19 trials with the device on (using Lanczos2 vision processing filter), and 2 trials with the device off. Due to the obvious floor effect with device off, there was no need to repeat this further. The patient wore a head-mounted camera that could be focused on different regions of the screen by adjusting her head azimuth or elevation. She was trained to find the center of each optotype by first judging it's width and height. From this central fixation point, the patient explored regions in each of the four possible directions, returning to the center each time and repeating until ready to respond. Descriptive analyses and the non-parametric Wilcoxon Rank Sum test were performed on the Landolt C data using Windows SPSS v22 (IBM Corporation, Somers, NY).

## Results

Three subjects with profound vision loss from retinitis pigmentosa consented for implantation; two males aged 49 and 63 years and a 52-year-old post-menopausal female. They were implanted with the suprachoroidal retinal prosthesis and percutaneous connector in mid-2012. Follow-up data were available for twelve months for each subject. Details of the clinical characteristics of subjects, electrode design/fabrication and surgical procedures are described in full in the methods.

**Figure 3:**
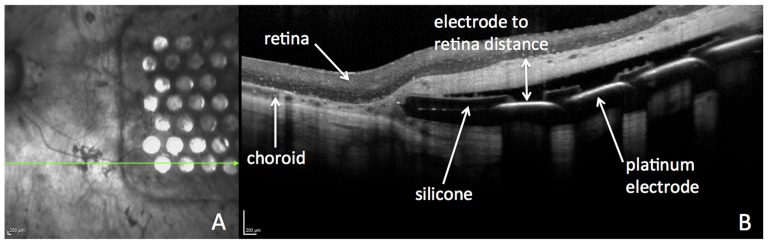
OCT scan of the electrode array in situ, taken 2 months postoperatively in Patient 1. The horizontal arrow on the infrared image indicates the direction of the OCT scan (A). The cross-sectional OCT image (B) shows the silicone and platinum electrode components of the array, the retina structure and choroidal vasculature, and the electrode to retina distance used for analysis (double-headed arrow). Scale bars  =  200 µm.

An optical coherence tomography (OCT) cross-sectional scan of the device *in vivo* is shown in Fig. 3. Due to the fact that the choroid becomes thinner in late stages of RP [22], the electrode array is easily visualized between the choroid and sclera.

### Surgical safety and efficacy

Implantation surgeries were performed under general anaesthesia and took three to four hours, with the duration decreasing between surgeries due to experience. The surgeries progressed without intraoperative complications. Due to the choroidal atrophy, the electrode array was easily sighted using conventional indirect ophthalmoscopy techniques during surgery, which allowed the device to be placed in the optimal position underneath the macula in all three cases. No intraocular or suprachoroidal hemorrhage was noted during the surgery.

### Postoperative clinical results: Retinal health

The postoperative recovery in all three subjects was similar to other major eye surgeries with respect to reported pain levels and the use of analgesia. Patient 1 (P1) reported more pain around the percutaneous plug implantation site (requiring narcotic analgesia with morphine 2.5 mg for the first two days) than P2 and P3, both of whom only required simple analgesia (oral paracetamol 1 g for the first two days). The level of intraocular inflammation was mild and similar to that seen in common procedures such as a retinal detachment repair, and treated with topical steroid (Prednefrin Forte, Allergan Australia, NSW, Australia) and antibiotic (Chlorsig, Aspen Pharma, NSW Australia) eye drops four times daily. There was no change in the intraocular pressure of the three participants over the duration of the trial, in either the implanted or control eye. One patient (P1) experienced mild to moderate limitation in the abduction of the implanted eye following surgery, but this function improved over the duration of the study and did not cause the patient any functional difficulty nor cosmetic concern.

**Figure 4:**
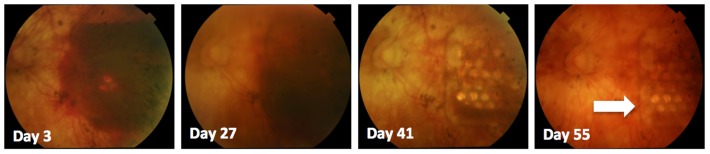
Time course of subretinal hemorrhage in P1, as documented with retinal fundus photography. Complete resolution of the hemorrhage occurred in this subject by 55 days post-operatively. Note the electrode array with individual electrodes can be seen more clearly over time as the blood clears in the temporal retina (arrow).

In all three subjects, a combined subretinal and suprachoroidal hemorrhage formed three to four days postoperatively, which resolved without any ongoing complications in P1 and P3. In P2, who experienced a larger hemorrhage than the others, a fibrotic tissue reaction remained at the temporal end of the implant following hemorrhage resolution, but this did not affect device efficacy nor cause complications such as retinal detachment. None of the subjects were taking anti-coagulant medication during this study. We did attempt to reduce eye movements in P2 and P3 by using intraoperative botulinum toxin injections into the rectus muscles and medications to reduce rapid eye movements during sleep, but the reduced eye movements did not prevent this hemorrhage from forming. Complete resolution of the hemorrhage took 55 days in P1, 101 days in P2 and 13 days in P3. Fundus photos showing the time course of the haemorrhage resolution for P1 are shown in Fig. 4. None of the subjects required any intervention during the resolution of the hemorrhage. Testing of the device did not commence until there was significant resolution of the blood, to allow clear visualization of the retina prior to any stimulation.

**Figure 5:**
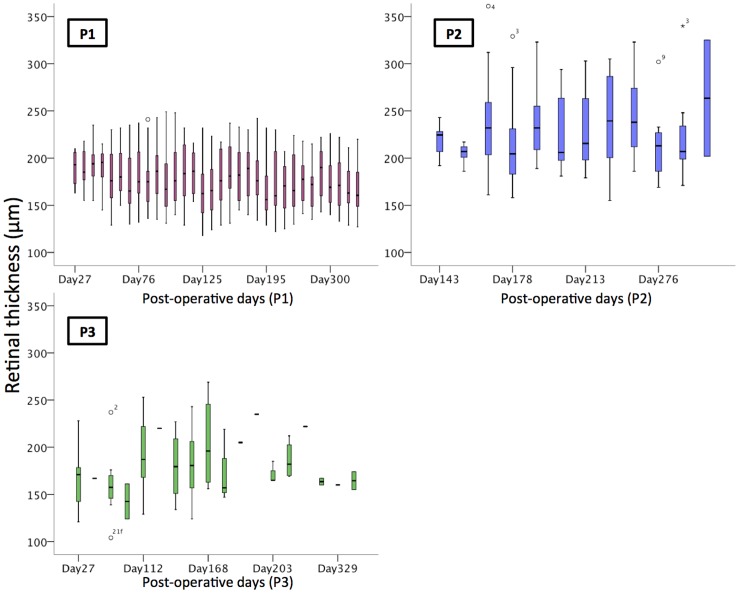
Retinal thickness measurements over time, showing no observable change in the maximum retinal thickness above the electrode array in the initial twelve months in all three patients. Each boxplot includes the maximum (upper whisker, excluding outliers), upper quartile (top of box), median (horizontal line in box), lower quartile (bottom of box) and minimum values (lower whisker). Open circles are outliers. The numbers represent the electrode location (see Fig. 2), and the horizontal lines on the graph of P3 represent single data points.

Longitudinal OCT measurements taken over the 12 month post-operative period demonstrated that there was no change in the retinal thickness above the electrode array, suggesting that no significant retinal oedema or atrophy had occurred after the implantation of the array (Fig. 5). Measurements of retinal thickness were taken directly above each of the 33 stimulating platinum electrodes, and the results shown reflect the data from all points. Note that as P2 and P3 had significant nystagmus, there was greater variability in the measurements due to decreased image quality and the fact that fewer locations were able to be sampled. Due to persistence of the subretinal and suprachoroidal hemorrhage in P2, retinal thickness measurements were not possible until Day 143.

### Postoperative clinical results: Summary of device-related serious adverse events

Subjects returned for post-operative assessment on a weekly basis during the entire study. The only device-related serious adverse event was a staphylococcus aureus infection around the percutaneous connector in P2 at day 59, which required a three-day hospital admission. The infection was successfully treated with a combination of intravenous vancomycin and flucloxacillin, and oral rifampicin and fusidic acid. The infection did not necessitate removal of the implant, nor did it have any effect on the stimulation or psychophysics testing. No device-related serious adverse events occurred with involvement of the intraocular electrode array.

### Postoperative clinical results: Device stability

**Figure 6:**
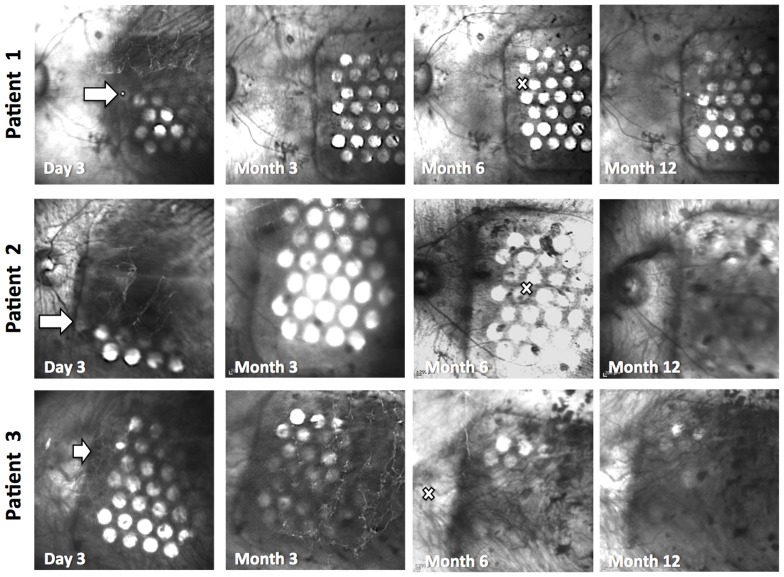
Time series of IR images of the electrode array in all subjects, showing no significant lateral movement of the array over a twelve-month period. The leading edge of the array is marked with a white arrow in the 3-day images. The position of the fovea is marked with a white cross in the 6-month images.

Intraocular array stability was monitored using fundus photography, infrared (IR) imaging and OCT. Measurements were taken of the position of the electrode array with respect to the optic nerve and vascular landmarks, and showed there was no lateral movement of the array with time. This stability can be seen in the time series of IR images shown in Fig. 6. Over time the individual electrodes became more difficult to visualize in P2 and P3.

The distance between the retina and the electrode array was measured from the cross-sectional OCT scans, and was taken from the anterior surface of the platinum electrode disc to the posterior surface of the retina (the retinal pigment epithelium, or RPE), as shown in Fig. 3.

**Figure 7:**
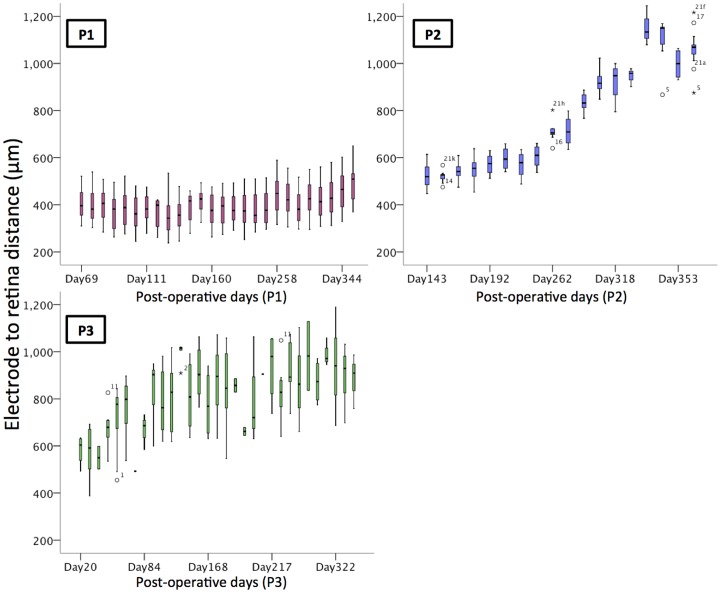
Distance between the electrodes and retina over time. This distance was relatively constant in P1, but increased up to two-fold in P2 and P3 over time. Note significant nystagmus in P3 made the measurements difficult, leading to greater variation in the values recorded. Outliers are identified by open circles, and the numbers represent the electrode location (see Fig. 2).

After the subretinal hemorrhage resolved, the electrode to retina distance has remained relatively constant for P1, but has increased for P2 and P3, Fig. 7. This increase in separation is visible on OCT scans, which show a hyper-reflective band between the choroid and the retina. The nature of this band is still under investigation.

**Figure 8:**
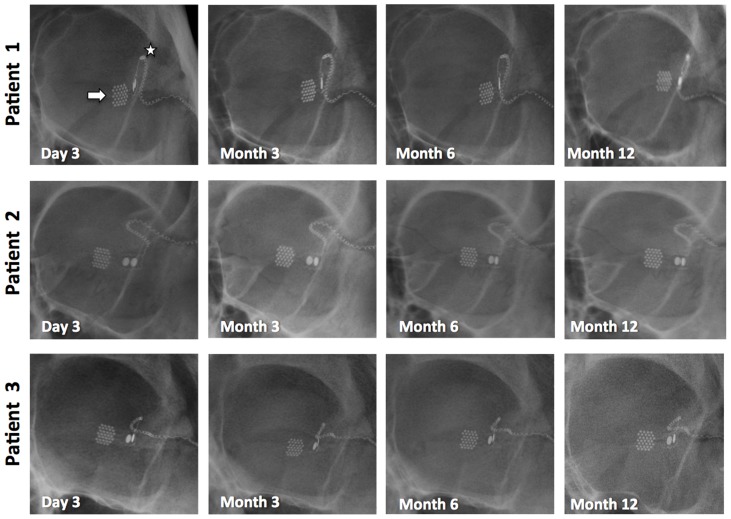
Stability of the intraocular array (arrow) and helical lead wire (star) over the initial 12 months of implantation, as documented by X-ray images. Additional scans were taken to monitor the percutaneous connector (not shown), which also stayed stable over this time period.

Stability of the titanium percutaneous connector and helical lead wire was monitored using monthly X-ray images. Comparison of the array and lead wire position was made with respect to anatomical landmarks, in three primary positions of gaze (straight ahead, left and right gaze). This also provided information about physical integrity of the lead wire, which showed no signs of stress or lead wire breakage. Over this initial twelve-month period, there was no significant movement of the intraocular array, lead wire or percutaneous connector on the X-ray images (Fig. 8). Note that the position of the zygomatic notch varies between subjects, hence there is variability in the vertical position of the lead entry point in the three subjects. The lead wire formed a broad superior loop in P1, and followed the temporal wall more closely in P2 and P3.

### Postoperative clinical results: Device integrity

Electrode channel continuity was verified by measuring intraoperative and post-operative electrode impedances. We measured impedance by applying a small test current pulse and measuring the electrode voltage. The resultant voltage was divided by the applied current to calculate impedance [40]. In all subjects, 100% of the electrodes within the implant remained intact following surgery and remained connected over the twelve-month period.

**Figure 9:**
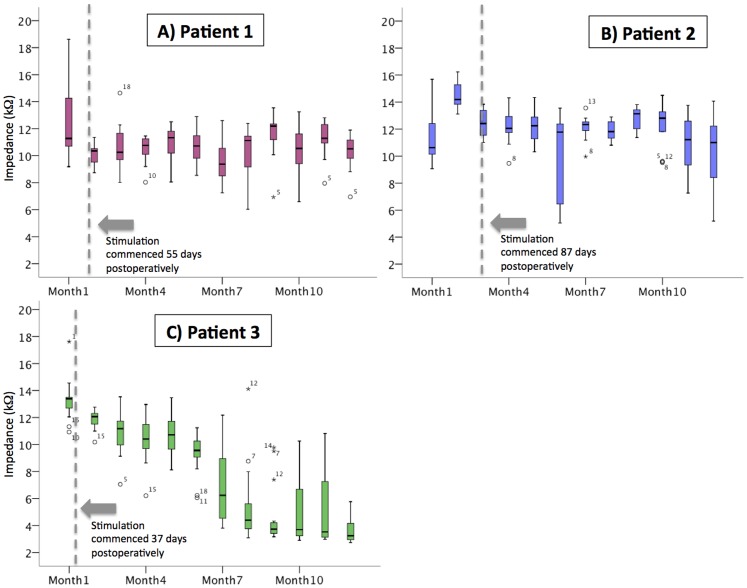
Impedances for the 600 µm platinum electrodes over time in the three subjects. Impedances were measured with charge-balanced biphasic current pulses (pulse phase width: 25 µs; amplitude: 75 µA). The dotted lines represent the date of first stimulation. In P1 & P2, the impedances were stable over the implantation and stimulation period. Impedances measured in P3 decreased over the course of the implantation period. Outliers are identified by open circles, and the numbers represent the electrode location (see Fig. 2).

Electrode impedance data were averaged monthly, from the date of implantation onwards, for each subject's array. The distribution of these data are plotted as box plots (Fig. 9A–C). The mean impedances for the 600 µm electrodes in P1 and P2 ranged over time from 9.7 to 14.5 kΩ and for P3 the mean impedance ranged over time from 3.6 to 13.3 kΩ. Impedance measurements were stable over the passive implantation and psychophysics testing periods in P1 & P2, but decreased significantly in P3 (Fig. 9C). This decrease in impedance suggests a change in the electrode- tissue interface or in the tissue surrounding the array. Further investigation continues to ascertain the exact mechanism of the decreased impedance for P3.

The range of measured impedances, particularly for P3, included some markedly low values. However, follow-up device integrity testing (electrode voltage tomography) on all three subjects did not reveal any evidence of short circuits in any of the electrode channels, suggesting that these low impedance values were genuine.

### Postoperative clinical results: Device efficacy (phosphene perception)

Following post-operative recovery and allowing time for almost complete resolution of the hemorrhage over the electrodes, stimulation of the electrode array commenced for weekly psychophysics sessions of between 2 and 5 hours. The first stimulation session was held 55 days postoperatively for P1, 87 days for P2 and at 37 days for P3. Stimulation was delivered using a custom-built neurostimulator (“neuroBi”), which allowed direct stimulation of the individual electrodes via connection with the percutaneous connector [27] and is designed to allow flexible configuration of testing parameters [41]. The stimulator delivered charge-balanced biphasic current pulses with pulse widths ranging from 100–1000 µs per phase. The electrodes were capacitively coupled and shorted between current pulses to remove any potentially damaging residual charge [42]. Unless otherwise stated, the electrodes were stimulated in a monopolar electrode configuration using one of the 2 mm diameter platinum electrodes as the return (see Fig. 2).

Reliable phosphene percepts were elicited in all three subjects. To ensure that overstimulation above safe charge and charge density levels did not occur, the charge per phase on each electrode was capped at 447nC for the 600 µm electrodes and 298nC for the 400 µm electrodes (which equates to an upper limit of charge density of 158 µC/cm^2^ and 237 µC/cm^2^). These values were determined using the Shannon model of safe levels for electrical stimulation, using a conservative *k* value of 1.85, as defined in Merrill et al [43]. Within this safe charge limit, percepts were able to be obtained on all 20 electrodes for P1 and 18/20 electrodes for P2 using monopolar stimulation at a rate of 50 pulses per second (pps). By increasing the stimulation rate to 500pps, percepts could be obtained from all 20 electrodes in P2.

**Figure 10:**
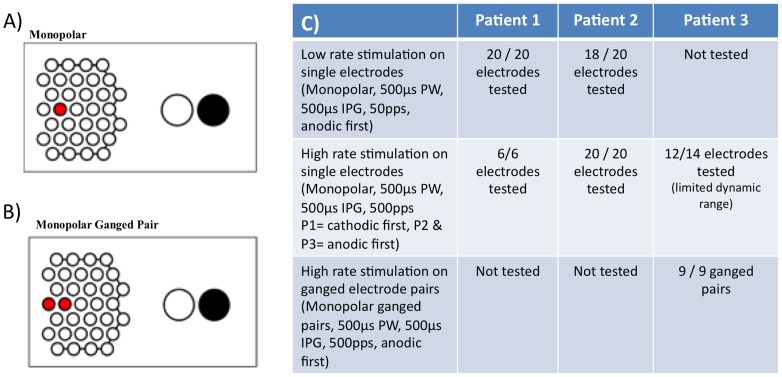
Different monopolar electrode stimulations used in the psychophysical testing of the implanted subjects (A & B), where the active electrodes are shown in red and the return electrodes in black. Table (C) shows the number of electrodes that were capable of eliciting a visual percept using the given stimulation parameters in each subject. PW =  phase width, IPG =  interphase gap, pps =  pulses per second. Stimulus duration was 2 seconds in all cases.

In P3, it was possible to elicit phosphenes using individual electrodes, however charge levels approaching the safe limit were required. Stimulation was subsequently performed using ganged pairs of adjacent electrodes as they provided greater dynamic range, allowing brighter percepts to be produced (Fig. 10B). Using ganged pairs increased the maximum safe charge per phase due to the larger total electrode area, thus enabling a 100% utilization rate for all ganged pairs tested with P3.

Phosphene appearance was found to be varied depending on subject, electrode position and stimulation parameters, but were controllable (in terms of size and, brightness and complexity), retinotopically placed and locatable in the visual field by the subject [44].

### Visual function results: Light localisation

In all subjects, the light localisation sub-task of the Basic Assessment of Light and Motion (BaLM) test [35] was completed to assess the device's ability to improve light localisation. In this test, the subject needs to identify the orientation of a wedge of light on a black computer screen (up/down/left/right), and it is a four alternative forced choice paradigm with a chance rate of 25%.

For the device on setting (using a Lanczos2 filter), all three participants performed significantly better than the 25% chance level. achieving 97.50% (*p*<.0001), 71.43% (*p*<.0001), and 66.66% (*p*<.0001) correct response rates for P1, P2 and P3 respectively. All of the participants performed at a level at or below chance when the vision processing system was switched off; Table 2.

**Table 2:** Results of the BaLM light localisation task, showing that using the device (with a Lanczos2 vision-processing filter) gave significant visual improvement compared with device off, p<0.0001 for all three subjects.

	Device On			Device Off			
Subject	Number of trials	Number correct	Percentage correct	Number of trials	Number correct	Percentage correct	Difference between device on and device off
P1	40	39	97.5%	72	20	27.78%	P<0.0001
P2	56	40	71.43%	40	10	25%	P<0.0001
P3	48	32	66.66%	40	10	25%	P<0.0001

### Visual function results: Optotype acuity estimates

**Figure 11:**
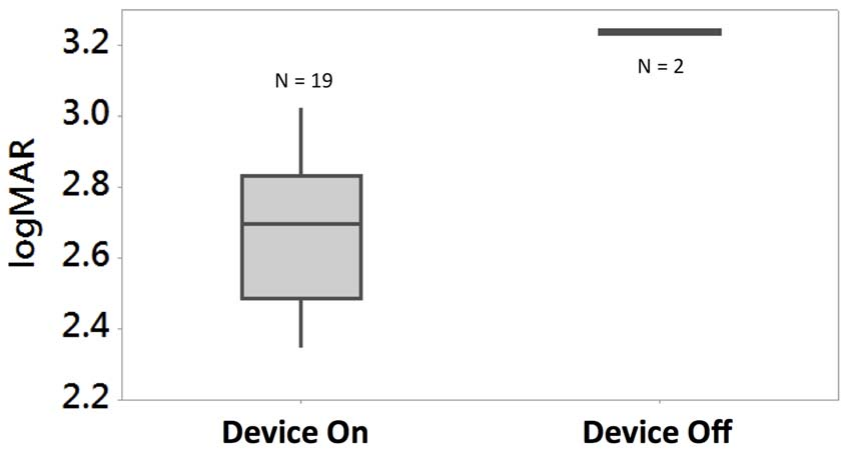
Optotype recognition results for P1 using the Landolt-C test, which gives an indication of acuity (but should not be directly interpreted as standard visual acuity). Whiskers extend to minimum and maximum recorded thresholds and the box extents show inter-quartile range. The Lanczos2 vision processing performed significantly better than System Off (P = .01). Note that the visual acuity exceeded the 3.24 logMAR software limit in all trials with device off (floor effect).

As P1 was implanted first, we have results for a longer post-implant time than for the other two participants. This enabled us to begin to measure optotype recognition using the Landolt-C task in the FrACT visual test battery [36]. For the device on setting (using the Lanczos2 filter), visual acuity was estimated to be 2.62 logMAR on average (range 2.35 to 3.02) corresponding to 20/8397 (range 20/4451 to 20/21059) over 19 sessions (Fig. 11). This compares favorably to the calculated limit of 2.33 logMAR (20/4242) for a 1 mm electrode pitch [37]. For the device off setting, P1 was unable to see any of the Landolt C optotypes at all. Due to the limit of the software (floor effect) we were unable to estimate any visual acuity lower than 3.24 logMAR. The average response time per optotype presentation was 59 seconds for device on (range 43 to 80 seconds) and 11 seconds for device off.

The non-parametric Wilcoxon Rank-Sum test was employed to determine whether there were differences between Lanczos2 vision processing and System Off in performance for visual acuity (logMAR) using Landolt C optotypes. Lanczos2 (*N* = 19, *Median* = 2.70) vision processing performed significantly better than System Off (*N* = 2, *Median* = 3.235) for P1 with *z* = −2.280, p = 0.010; Fig. 11].

## Discussion

This world-first Phase 1 trial of a retinal implant in the suprachoroidal space has shown that the novel surgical procedure is safe, the position is stable and the device was able to provide visual percepts in all three subjects. The electrode array was well tolerated by the eye and all electrodes on the three arrays remained functional over the 12-month monitoring period.

The main advantage with the suprachoroidal surgical approach is the ease and safety of the surgical procedure, as well as the proven device efficacy in all three of our subjects. It is remarkable that all electrodes remained functional for our subjects for the 12-month period, as this has not been the case with previous devices. For example, a report to the US Food and Drug Administration in September 2012 reported that in the Argus II 60-electrode device clinical trial, an average of 55.5±3.6 electrodes were functioning directly after implant, with another 1.2±1.8 electrodes automatically disabled during stimulation due to high impedances [20].

The implantation surgery for each device took less than four hours, and there were no intraoperative adverse events in any subject. It is anticipated that the surgical procedure used for the implantation of these suprachoroidal devices could be undertaken by the majority of ophthalmic surgeons, which to date has not been the case for the other retinal approaches. It is also anticipated that this location may allow for simpler removal and replacement with upgraded devices in the future. This has been demonstrated in preclinical feline models [45] but not yet in humans.

The devices have been mechanically stable over the initial postoperative twelve months, with no lateral movement (with respect to the retinal vasculature landmarks) nor signs of extrusion. The one serious device-related adverse event that did develop in this initial twelve-month period was an infection of the percutaneous connector, which was an anticipated risk (having been noted in previous trials using percutaneous connectors for cochlear implant research) and was successfully treated with intravenous antibiotics with no damage to the device, nor need for explantation. The use of a percutaneous connector allowed direct and flexible stimulation of the intraocular electrode array, but required a metallic foreign body to protrude from the skull for the duration of the study. In subsequent studies it is planned that the percutaneous connector will be replaced with a fully implantable and wireless stimulator system, hence eliminating the risk of such infections in future iterations.

The other adverse event that was noted was that all three subjects had a combined subretinal and suprachoroidal hemorrhage three to four days post-operatively, which resolved without complication in two patients. In P2, a small fibrovascular scar remained at the edge of the implant following haemorrhage resolution, which has been stable for 12 months. After noting this in our first subject, the study protocol was changed to include intraoperative botulinum toxin injection and post-operative oral medication to reduce rapid eye movement during sleep, in the hope that a more stable eye during the initial healing period would prevent the formation of this hemorrhage. However, this was not the case, and the exact mechanism of the hemorrhage remains unclear.

Due to the suprachoroidal implant location, we were concerned about the risk of damaging the long posterior ciliary neurovascular bundle during implantation, as this bundle pierces the sclera and crosses the suprachoroidal space [46]. We were prepared for the risk of contacting this bundle with a surgical contingency plan, which included methods of diathermy to stem any bleeding, as outlined previously [33]. However, this was not required in any of the patients. Due to the fact that the postoperative bleeding originated in the anterior choroid (not in the suprachoroidal space) and occurred in the postoperative period, we do not believe that this was the cause of the observed hemorrhages. However, the presence of the long posterior ciliary neurovascular bundle does need to be considered during suprachoroidal implant surgeries.

As can be seen in the IR images (Fig. 6), the electrodes have become more difficult to visualize with time, especially in P3. This is correlated with an increase in the electrode to retina distance in P3. This may be due to a change in the tissue-electrode interface, but due to limitations in ocular imaging techniques associated with the subject's nystagmus, we cannot conclusively determine whether this area is fibrous tissue, fluid or some other material. Ongoing investigations are continuing, and it is likely that we will need to wait until explantation of the devices to gain further information. This outcome was not noted in our preclinical feline models.

Intra-operative and post-operative impedance testing demonstrated ongoing electrode continuity over a twelve-month follow-up period. These results suggest a robust electrode array, lead system and connector. This is a significant finding, as it is very common for some electrodes and even the lead wires to break during implantation surgery in other locations. In addition to demonstrating surgical safety, the fact that all electrodes within the clinical arrays were viable after twelve months implantation shows the mechanical robustness and stability of the suprachoroidal position and associated lead routing path.

Another interesting finding has been the decrease in impedances in P3 over time. Whilst P1 and P2 maintained a mean impedance of 9.7–14.5 kΩ over the study period, impedances for P3 dropped from an initial mean measure of 13.3 kΩ to a mean of 3.6 kΩ by twelve months. A drop in impedance suggests a change in the electrode-tissue interface or the tissue surrounding the array, but the exact cause for these changes are not known, with further investigations underway to ascertain the potential mechanism.

The impedance results are comparable to those derived from previous studies in our feline model, using a similar suprachoroidal position for the electrode array with platinum electrodes of the same dimensions [40]. These preclinical studies showed stable mean impedances in the range of 11–15 kΩ over a 5 month implantation period with approximately three months of continuous suprathreshold electrical stimulation in the suprachoroidal space. The similarity between impedances measured clinically and preclinically adds support to the veracity of the feline suprachoroidal model published previously by our group, which will be used for ongoing development of future devices.

We were able to generate visual percepts in all three subjects within a safe charge limit. It was necessary to use ganged pairs of electrodes to gain useful percepts with a satisfactory dynamic range for P3, which may be due to the increased distance between the array and the retina, or due to the array being further from the macula than in the other two subjects (see Fig. 6). Unlike the other two participants, P3 is also on anti-epileptic medication (valproate sodium 100 mg), which potentially may have a role determining the parameters needed to record a percept. Further details on the factors that affect phosphene perception in this cohort are detailed in a separate publication by Shivdasani et al [47].

As this was an initial proof of concept study, with only three participants were recruited, it was not possible to draw inferences about the effect of gender, age, surgical ancillary treatment (i.e. the use of Botox injections during implantation) or systemic medications on the outcomes. It is certainly of interest that the results varied between our three participants, and this gives support to our belief that device fitting and outcome assessment for bionic eyes will remain a time-consuming and individual process.

Device efficacy was also shown using a test of light localisation (BaLM test) and a Landolt-C optotype recognition test (FrACT test). Light localisation is a simpler visual function than optotype recognition, but does not enable an estimate of visual acuity. We demonstrated that all subjects showed improved light localisation with the device on (all passing the test's “pass” criterion of 62.5%), with performance with device off below the level of chance. We found that the Landolt-C test required significant orientation and habituation, which was not possible in the 12-month postoperative time frame for P2 and P3. As P1 was implanted first, we were able to estimate her optotype acuity using this test, and showed that it was significantly improved with device on (best logMAR measured = 2.35) vs. device off (logMAR worse than 3.24, reached the floor effect of the test). Previously reported visual acuity from other groups have been up to 20/1262 (logMAR 1.8) for the Argus II epiretinal device (60 electrodes) using grating acuity tests [15] and 20/546 (logMAR 1.43) for the Retina Implant AG Alpha IMS device (1500 independent microphotodiode-amplifier-electrode elements) using the Landolt C optotype acuity test [48].

It should be noted that the use of Landolt C optotype recognition tests in prosthetic vision gives estimations of acuity only, and cannot be directly compared with acuity tests in normally sighted individuals. Subjects with a visual prosthesis will use different techniques to assess the optotypes, including head scanning and learnt interpretation of the phosphenes. Thus the visual acuity reported using visual prostheses is likely to be overestimated. Future studies will be vital to obtain more detailed information regarding the potential visual acuity of the suprachoroidal devices in a greater number of participants, and this estimated acuity value should be interpreted with caution, and whilst remembering that our prototype device only had 20 stimulating electrodes.

This study has shown the suprachoroidal anatomical position is a viable, minimally invasive and relative straightforward location for an electrode array, with successful stimulation of visual percepts and excellent lateral device stability in all subjects. The device efficacy is expected to improve with technological advances in the next iteration, and also with the ability for patients to be trained on use in their home environment in future trials (which was not possible in this study). We will continue to investigate the presence of the band between the device and choroid, including using histopathological assessment techniques on explanted devices at the end of this study (which will cease in late 2014). The inherent advantages of the suprachoroidal position, including ease of surgery (no vitrectomy or retinal incision required), robustness and stability of the electrodes, and minimal implant-related intraocular serious adverse events suggest that this approach has promise in the field of retinal prosthesis research.

## Supporting Information

S1 TREND Checklist **TREND Checklist.**
(PDF)

S1 Protocol **Trial Protocol.**
(PDF)
