# Supplementary material for: First-in-Human Trial of a Novel Suprachoroidal Retinal Prosthesis
Source: PLoS One. 2014 Dec 18;9(12):e115239. doi: 10.1371/journal.pone.0115239 (PMC4270734; doi:10.1371/journal.pone.0115239)
Supplement: S1 Protocol — Trial Protocol. (PDF) [file pone.0115239.s002.pdf]

Study Protocol BVA_24ChPP:
24 Channel Percutaneous Plug Study

Sponsor:	**Centre for Eye Research Australia (CERA)**
Authoriser on behalf of the Sponsor:	Professor Jonathan Crowston

Principal Investigator:	Professor Robyn Guymer
Trial Site:	**Centre for Eye Research Australia (CERA)**<br>Level 1, 32 Gisborne Street, East Melbourne VIC<br>Ph. (03) 9929 8360

Other involved institutions:	**Bionics Institute (BI)**<br>384 – 388 Albert St, East Melbourne VIC 3002<br>Ph. (03) 9667 7500<br><br>**Royal Victorian Eye and Ear Hospital (RVEEH)**<br>32 Gisborne St, East Melbourne VIC 3002<br>Ph. (03) 9929 8666

Investigators Signature Page

I Robyn Guymer agree to conduct this clinical trial according to the declaration of Helsinki (2000), International Conference on Harmonisation Good Clinical Practice (ICH-GCP) and to abide by the protocol 24Ch:4.

The Centre for Eye Research Australia (CERA) will conduct the study in strict compliance with this protocol.

On behalf of CERA [signature] , *Prof Robyn Guymer*

Table of Contents

Investigators Signature Page 1
Abbreviations and Definitions of Terms 4
1. Synopsis 6
2. Introduction 8
2.1. Summary of known and potential risks and their severity 8
2.2. Description of trial population 10
2.3. Description and justification of implant period 10
2.4. Literature review 10
3. Trial Objectives and Purpose 11
4. Trial Design 12
4.1. Primary and secondary endpoints 12
4.2. Participant screening and recruitment 12
4.3. Post-surgery trial design 15
4.4. Discontinuation criteria 15
4.5. Data collection procedures 15
5. Trial Population 15
5.1. Number of subjects 15
5.2. Inclusion criteria 15
5.3. Exclusion criteria 16
6. Trial Assessments and Procedures 16
6.1. Screening evaluation 16
6.2. Trial procedures 18
7. Subject Completion and Discontinuation 20
7.1. Subject completion 20
7.2. Stopping rules / Discontinuation criteria 20
7.3. Subject withdrawal 20
7.4. Early termination of the study 20
8. Case Report Form (CRF) 21

9. Data Analysis and Statistical Considerations 21
10. Data Management 22
11. Monitoring and Quality Assurance 22
11.1. Curriculum Vitae and other documentation 23
12. Investigator Responsibility 23
13. Study Report 23
14. Administrative Procedures 23
14.1. Ethical considerations 23
14.2. Ethical review committee 24
14.3. Regulatory authorities 24
14.4. Informed consent 24
14.5. Subject reimbursement 24
14.6. Emergency contact with investigators 25
14.7. Notification of primary care physician 25
14.8. Investigator indemnification 25
14.9. Financial aspects 25
14.10. Protocol amendments 25
14.11. Protocol compliance 26
14.12. Archives: Retention of study records 26
15. References 27

Abbreviations and Definitions of Terms

24ChPP	24 channel percutaneous plug
AE	Adverse Event
BI	Bionics Institute (the manufacturer of the WPCD device and NeuroBI stimulator, and location of psychophysical test sessions)
BVA	Bionic Vision Australia (consortium of research institutes and universities working to develop a bionic eye)
CERA	Centre for Eye Research Australia (the Sponsor and site of this study)
CIB	Clinical Investigators' Brochure
CRF	Case Report Form
CTN	Clinical Trial Notification
ERG	Electroretinogram
GCP	Good Clinical Practice
HREC	Human Research Ethics Committee
ICH	International Conference on Harmonisation
IEC	Independent Ethics Committee
NHMRC	National Health and Medical Research Council
NICTA	National ICT Australia (a BVA partner institution)
O&M	Orientation and mobility
Perc	Percutaneous
PI	Principal Investigator
PICF	Patient Information and Consent Form
PP:C:1	Abbreviation for SOP #1 for the perc plug study (PP), from the clinical team (C)
PP:PT:1	Abbreviation for SOP #1 for the perc plug study (PP), from the psychophysics

	team (PT)
PP:S:1	Abbreviation for SOP #1 for the perc plug study (PP), from the surgical team (S)
RP	Retinitis Pigmentosa
RVEEH	Royal Victorian Eye and Ear Hospital (location of implant surgery, responsible for ethical approval and provision of clinical trials insurance)
SAE	Serious Adverse Event
SOP	Standard Operating Procedure
TGA	Therapeutic Goods Administration
WPCD	Wide view bionic eye percutaneous device

1. Synopsis

Study Title: Evaluation of a supra-choroidal retinal prosthesis: A 24 Channel percutaneous connector study

Protocol Number: BVA_24ChPP **CTN Number:** 090/2012

RVEEH Human Research Ethics Approval Number: 11/1032H (approved September 2011)

Indication: Retinal visual prostheses are currently in early development as an intervention to improve functional vision in people who have become blind from retinal degenerative eye disease.

Investigational device: A 24 channel percutaneous plug connected wide-view retinal prosthesis

No. of subjects: 3 **No. of centres:** 1

Study duration: 24 months

Objectives of the study:

- Primary = pilot study aimed at evaluating components of the device, gaining experience with implanting the device and learning how visual data can be provided to recipients to create perception.
- Secondary = enhance the device design, surgery and stimulation strategy for the next generation retinal prostheses being developed by Bionic Vision Australia

Study endpoints:

- Primary = safe implantation and removal of investigational device in 3 human subjects
- Secondary = development of a "phosphene map", a collection of psychophysics data that will inform the visual processing in future bionic eye devices, and help us to understand how visual data can be provided to recipients to create perception

Study design: Interventional prospective study

Study procedures:

- Surgical implantation of investigational device
- Direct electrical stimulation of the implanted retinal prosthesis using a percutaneous connector
- Psychophysical testing of patient thresholds and perceptions when using varying electrical stimulation patterns
- Retinal imaging
- Ocular health assessments to ensure stable retinal health throughout the study
- Visual function and functional vision assessments

Eligibility criteria:

Inclusion criteria	Exclusion criteria
• Aged 18 years or older<br>• Either gender<br>• A confirmed history of outer retinal degenerative disease such as retinitis pigmentosa or choroideremia<br>• Remaining visual acuity of bare light perception or less in both eyes<br>• Functional inner retina (ganglion cells and optic nerve), as shown by the ability to perceive light and/or a measurable corneal electrically evoked visual response<br>• A history of at least 10 years of useful form vision in the worse seeing eye<br>• Must be willing and able to comply with the testing and follow-up protocol demands (preferably residing within 1.5 hours of the investigational site)	• Optic nerve disease (history of glaucoma of more than 1 month, or history of any other optic neuropathy)<br>• Diseases of the inner retina including, but not limited to, central retinal artery or vein occlusion (CRAO, CRVO), end stage diabetic retinopathy, retinal detachment, traumatic retinal damage, infectious retinal disease, inflammatory retinal disease.<br>• Inability to visualise the retina due to corneal or other ocular media opacities (corneal degenerations, dense cataracts, trauma, lid malpositions)<br>• Any ocular condition that predisposes the subject to rubbing their eyes<br>• Cognitive deficiencies, including dementia or progressive neurological disease<br>• Psychiatric disorders, including depression, as diagnosed by a qualified psychologist<br>• Deafness or significant hearing loss<br>• Inability to speak or understand English<br>• Pregnancy<br>• Presence of a cochlear implant<br>• Subject enrolled in another investigational drug or device trial for the treatment of their ocular condition<br>• Poor general health, which would exclude them from obtaining a general anaesthetic<br>• Unrealistic expectations of the investigational device to provide functional vision

2. Introduction

Bionic Vision Australia is currently developing a retinal prosthetic device (also known as bionic eye) for vision restoration in patients with photoreceptor degenerative diseases such as retinitis pigmentosa (RP) and choroideremia. The device will be implanted in a space between the sclera and the choroid, called the suprachoroidal space, and positioned behind the macular area. This suprachoroidal approach has several advantages over other intraocular including a simplified surgical procedure, less surgical complications such as a retinal detachment, and more importantly, the implant is more mechanically stable.

Data from pre-clinical animal experimental studies shows that it is feasible and safe to implant an electrode array into the suprachoroidal space. Furthermore, electrical stimulation of the retina using the implanted electrode array activates responses from the visual cortex suggesting that the brain can "see" when the implanted electrode is stimulated. We have also completed preclinical studies that show that the suprachoroidally implanted electrode array can be removed from behind the retina with no discernible damage to the eye, allowing for electrode extraction when required. A non-active electrode has been implanted in an acute study in a human prior to pre-planned enucleation surgery, but has not yet been implanted or stimulated chronically in humans.

The objective of this pilot study is to evaluate in humans the components of the Bionic Vision Australia suprachoroidal electrode array, gain experience with the surgical technique and optimise the computation and transfer of visual data. This device will be stimulated via a percutaneous connector, which allows our researchers to directly stimulate the electrodes in a laboratory environment. Each electrode will excite surviving nerve cells to create artificial percepts. Each electrode within the array will be wired directly to a pin on a single connector that protrudes through the skin so that electrical stimulation can be applied to the electrode array and explore the resulting visual percepts.

2.1. Summary of known and potential risks and their severity

Risks are listed in order of likelihood (likely, unlikely, and extremely unlikely) and estimated severity of each risk is denoted in italics. A full risk assessment has been undertaken for this study, and can be found in the BVA_24ChPP Trial Master File and in the GCP SOP 6.4.

Likely

- Redness and irritation of the eye in the early postoperative period (2- 3 weeks). *Mild to moderate severity.*
- Mild pain on eye movement in the early postoperative period (2- 3 weeks). *Mild severity.*
- Mild scarring where the incisions for the lead wire and percutaneous connector are made at the corner of the eye and behind the ear. *Mild severity.*
- Mild bruising near the site of injections or drip lines. *Mild severity.*
- Fatigue during testing sessions. *Mild severity.*

- Subretinal haemorrhage in the early post-operative period (up to one week). *Mild to moderate severity.*

Unlikely

- Infection of the implant or of the eye. *Mild to severe.*
- Infection of the external wound site (around the connector). *Mild to severe.*
- Damage to the electrode array and/or lead wire when the device is implanted. *Mild to severe.*
- Reaction to the post-surgical drugs (i.e. antibiotics, steroids). *Mild to severe.*
- Psychological reaction to the surgery and testing protocol- depression, anxiety etc. *Mild to severe.*
- Short-term increased intraocular pressure after surgery (which may cause damage to the optic nerve). *Mild.*
- Scarring around the implant and lead wire. *Mild to severe.*
- No electrodes work when the device is connected to the stimulating equipment. *Mild.*
- Over-stimulation of the electrodes, so that you would feel the electrode being turned on and off, or would experience a very bright light sensation. *Mild to severe.*
- Some electrodes produce a painful sensation when stimulated. *Mild to Severe depending on the number of electrodes eliciting pain.*
- Failure to remove the electrode array when the study is completed. *Mild to severe.*
- The need for fluorescein angiography may be indicated and if performed, there are a number of risks that may occur. The injection of the dye may turn skin a yellowish colour for several hours until it is filtered out of the body by the kidneys. As it is filtered out, it will also turn urine a dark orange colour for up to 24 hours following the test. Some people can experience nausea during the procedure, but this usually passes quickly, within a few seconds. If the dye leaks out of any of the blood vessels, localized burning and staining of the skin may occur. The burning will only last a few minutes, and the yellow staining can last for a few days. More severe reactions such as allergy to the dye or anaphylactic shock are extremely rare, and patients will be monitored closely for signs of these potentially life-threatening conditions. *Mild to severe.*

Extremely unlikely

- Extrusion or rejection of the implant from the eye. *Severe.*
- Damage to the retina including, but not limited to, retinal detachments, retinal haemorrhages, or optic nerve damage. *Moderate to severe.*
- Reactions to the general anaesthetic, ranging from mild nausea to death or disablement. *Mild to severe.*
- Damage to the external structures of the eye and orbit, including damage to the muscles, tissues, nerves or blood vessels surrounding the eye. *Mild to severe.*
- Damage to the facial nerve, which may lead to facial paralysis. *Mild to severe.*
- Long term pain or discomfort. *Mild to severe.*
- Loss of the eye due to infection, inflammation or traumatic damage. *Severe.*
- Electrical shock. *Mild to Severe.*

- Damage to the non-implanted eye, due to an inflammatory response known as sympathetic ophthalmia. This can be treated with steroids, but very rarely will lead to enucleation and loss of the untreated eye. *Mild to severe*.

2.2. Description of trial population

Three individuals with severe visual loss (bare perception of light or worse) from retinal degenerative diseases such as RP, each of whom will then be followed for 2 years for measurements of device efficacy and stability. The population are over the age of 18 with a history of at least 10 years of useful form vision in the worst seeing eye.

2.3. Description and justification of implant period

As a pilot study our patient population for these early trials is small, thus we believe it is important to gain as much useful information from each implantation as possible. It is predicted that the patients will need a period of at least a month to fully recover from the surgery followed by an adaptation/learning period of unknown length to provide consistent repeatable data. Thus a two-year project has been designed.

2.4. Literature review

Blindness is one of the most feared disabilities,[1] having a significant and debilitating impact on both an individual's quality of life and the wider social economy. The most common genetic cause of blindness associated with the loss of retinal photoreceptors is retinitis pigmentosa (RP). Worldwide, there are about 1.5 million people who suffer from RP, which makes it the leading cause of inherited blindness.[2]

There is no cure for RP at this time. Many people have already lost their sight to this condition, and it is for these people that the visual prosthesis (bionic eye) may restore some functional vision. Visual prostheses work to convert visual information into electrical impulses, in a similar way that cochlear implants have worked to restore hearing to the deaf. Research is underway in a number of centres around the world into various forms of visual prostheses, such as retinal (suprachoroidal, sub-retinal and epi-retinal implants), optic nerve cuffs and cortical implants. The majority of groups, including Bionic Vision Australia (BVA), are investigating the retinal approach. While the theory behind retinal visual prostheses has been proven by clinical trials in the USA and Germany,[3,4] it is still undeniable that the development of a bionic eye is one of the most difficult technological challenges that biomedical engineering has faced to date.

For a retinal implant to be effective, sufficient inner retinal neurons must remain despite severe photoreceptor cell loss in degenerative retinal disease. A recent frequency-domain optical coherence tomography study by Hood et al revealed that this is the case in RP, with no significant difference between the thickness of inner nuclear and retinal ganglion cell layers in RP patients and controls.[5] Hence,

theoretically, retinal micro-electronic implants should allow some level of vision in patients who are blind from this condition, as there is limited trans-synaptic neurodegeneration.[6] In other words, the retinal prosthesis works by bypassing the dead photoreceptors to directly stimulate the inner retinal neurons, thus utilising the intact posterior visual pathway to transmit signals to the visual cortex.

It has been shown that retinal visual prostheses placed on the retina[4,7] (epi-retinal implant) or between the retinal layers[3] (sub-retinal implant) can both evoke phosphenes, or perception of light spots, in patients that are blind from RP. It has been shown that trans-choroidal electrical stimulation can evoke phosphenes in humans[8], but to date there have been no human tests of electrode arrays implanted within the suprachoroidal space. The suprachoroidal space is an area between the white of the eye (the sclera) and the posterior blood supply (the choroid). The advantage with this location is that there is increased stability in this location, and there also should be less chance of surgical complications to the retina itself.

Our research team has shown the efficacy of suprachoroidal visual implants in pre-clinical animal studies.[9-13] Our current work has also shown that the electrode array can be removed safely from the suprachoroidal space (Leung et al., unpublished data 2011). However, there have been no chronic tests of the suprachoroidal implant to date in humans.

Percutaneous connectors have been used previously in cochlear implant research,[14] and allow researchers to safely control the amount and timing of electrical stimulation to the array before the electronics are designed to be implanted. It is an intermediate step which allows the investigation of the stimulus parameters that are required to elicit phosphenes with the electrode array, but before all steps are completed for the fully integrated device.

3. Trial Objectives and Purpose

The wide-view percutaneous connector study is an essential step in the development of the wide-view bionic eye device by Bionic Vision Australia (BVA).

The goals are to conduct research and provide proof of concept in blind patients with retinitis pigmentosa for several core aspects of the wide-view device including electrode placement in the suprachoroidal space, enhance the device design and collect psychophysics data to inform the development of visual processing techniques.

If successful, this project will lead to an implantable wide-view prototype device suitable for a clinical trial aimed at improving mobility and orientation in people with advanced retinitis pigmentosa within the next two years, with much reduced clinical, technical, and commercial risks. Improved mobility and orientation will lead to increased independence, self-confidence, and quality of life in the device recipients.

4. Trial Design

The study will be broken into three phases:

Phase 1, which will last approximately 2 months with each patient, is designed to utilise the surgical technique and assess and describe the visual percepts produced by electrical stimulation of suprachoroidal electrodes in blind humans.

Phase 2, which will last for up to another 16 months with each patient, is designed to obtain detailed human psychophysical data for the suprachoroidal implant. We will aim to collect data on more detailed questions about how to describe, control, and optimise the visual percepts produced by electrical stimulation of the retina.

Phase 3, which will form the final 12 – 24 months of the study, is designed to enable assessment of "real-world" situations by connecting the intraocular electrodes to a camera and semi-portable vision processor. This extension to the study will enable researchers to measure the real world acuity and functional vision performance with this prototype device.

4.1. Primary and secondary endpoints

Primary = safe implantation and removal of investigational device in 3 human subjects

Secondary = development of a "phosphene map", a collection of psychophysics data that will inform the visual processing in future bionic eye devices, and help us to understand how visual data can be provided to recipients to create perception

4.2. Participant screening and recruitment

Research subjects will be identified from a concurrent research project being completed at CERA (RVEEH HREC09/921H): Structure function relationship of the inner retinal layers in eyes with profound loss of photoreceptors.

If a person is deemed a potential candidate for the BVA_24ChPP study from the structure-function study results, they will be invited by phone to return for further testing (the perc plug screening visit and the functional vision/orientation and mobility home assessment). At this time, a decision on eligibility will be made based on the selection criteria. If the person is eligible and potentially interested in being involved in the research study, they will be asked to attend an information session with the PI and senior research staff (Prof Robyn Guymer, Dr Penny Allen, Prof Peter Blamey, Dr Chi Luu, Dr Lauren Ayton), at which time all details of the study will be discussed and the participant will be given copies of the participant information and consent form (PICF) in their preferred modality (written, audio or electronic).

A senior investigator will contact the participant by phone within one week to discuss further and, if the patient is interested in proceeding, a visit to obtain signed informed consent will occur. The following flow chart details the steps taken during patient recruitment and screening.

VISIT ONE (Structure- Function Study, Ethics approval RVEEH HREC 09/921H)

The results of Visit 1 will enable investigators to decide whether the participant retinal structure and function are appropriate for this study, in order to identify those who can return for further screening visits. Basic psychological screening and general health observations will be made at this initial visit, but the main aim is to categorise disease severity. *CRF: Structure function study data form*

VISIT TWO (BVA_24ChPP study screening visit)

The study screening visit will involve replication of the structure/function visit assessments and more detailed psychological and general health screening tests. Participant aims/motivations will be evaluated at this time, to gauge interest in the study. Eligible participants who are interested in the study will be invited to an information session about the project . *CRF: Perc plug screening visit data form*

VISIT THREE (BVA_24ChPP study functional vision assessment)

An assessment of the participant's functional vision will be made in the hospital and/or in the participants home environment. This will include assessment of orientation and mobility and activities of daily living.

VISIT FOUR (BVA_24ChPP study information session)

The participant will meet with key research team members at this stage (i.e. surgeons, heads of psychophysics programs etc), to discuss the study in detail. If possible, a family member or friend should attend this visit. After the session, participants will be given electronic and/or audio copies of the PICF to consider in their own time. An investigator will contact the participant within one week to answer any questions and determine whether participant wishes to be involved in the study.

VISIT FIVE (BVA_24ChPP informed consent and psychological screening visit)

At the fifth visit, signed written informed consent will be obtained. Remaining tests from the psychological screening battery will be completed. *CRF: Psychological screening data form*

VISIT SIX (BVA_24ChPP independent psychologist assessment)

The sixth visit will be an assessment by an independent psychologist, who will assess the participant for psychosocial disorders, depression and other psychological disorders. They will specifically seek to assess whether the participant has been properly consented to the study (ie. if they have a good understanding of the project), and to provide an independent source of support to the participant.

VISIT SEVEN (BVA_24ChPP general health examination and baseline visit)

This visit will occur in the two weeks prior to implant surgery. A general health examination will be performed by the surgeon and anaethetist to ensure suitability for surgery. This will involve standard medical assessments, including imaging.

At the same visit, baseline data will be collected for visual function and ocular imaging, to be used for comparison post-surgery.

4.3. Post-surgery trial design

Approximately one month after the implant surgery (depending on healing times), participants will come to the trial site for 8 hours of psychophysics testing per week, for up to 2 years. Participants will be given the option to attend for one or two days, depending on their personal availability and needs. For full details of the trial procedures, see section 6.2.

In Phase 3, which is the extension to the original study to allow testing of "real-world" functional vision with a camera and semi-portable vision processor", participants will be given the option to attend for one or two days per week, up to a maximum of 16 hours testing per week.

4.4. Discontinuation criteria

The trial will be discontinued if there is significant harm or risk to patient safety, as assessed by the head surgeon, or if the device is not working under any stimulation conditions.

4.5. Data collection procedures

Data will be collected on case report forms, which will be stored in the study file. At the completion of each phase of testing, the CRFs will be scanned and the electronic versions stored on the secure MRU server.

Specific data collection procedures are included in the SOPs for each test.

5. Trial Population

5.1. Number of subjects

Three

5.2. Inclusion criteria

- Aged 18 years or older
- Either gender
- A confirmed history of outer retinal degenerative disease such as retinitis pigmentosa or choroideremia
- Remaining visual acuity of bare light perception or less in both eyes
- Functional inner retina (ganglion cells and optic nerve), as shown by the ability to perceive light and/or a measurable corneal electrically evoked visual response
- A history of at least 10 years of useful form vision in the worse seeing eye

- Must be willing and able to comply with the testing and follow-up protocol demands (preferably residing within 1.5 hours of the investigational site)

5.3. Exclusion criteria

- Optic nerve disease (history of glaucoma of more than 1 month, or history of any other optic neuropathy)
- Diseases of the inner retina including, but not limited to, central retinal artery or vein occlusion (CRAO, CRVO), end stage diabetic retinopathy, retinal detachment, traumatic retinal damage, infectious retinal disease, inflammatory retinal disease.
- Inability to visualise the retina due to corneal or other ocular media opacities (corneal degenerations, dense cataracts, trauma, lid malpositions)
- Any ocular condition that predisposes the subject to rubbing their eyes
- Cognitive deficiencies, including dementia or progressive neurological disease
- Psychiatric disorders, including depression, as diagnosed by a qualified psychologist
- Deafness or significant hearing loss
- Inability to speak or understand English
- Pregnancy
- Presence of a cochlear implant
- Subject enrolled in another investigational drug or device trial for the treatment of their ocular condition
- Poor general health, which would exclude them from obtaining a general anaesthetic
- Unrealistic expectations of the bionic eye device

6. Trial Assessments and Procedures

6.1. Screening evaluation

Participants will be initially screened in another CERA study – Structure-Function Relationship of the Inner Retinal Layers in Eyes with Profound Loss of Photoreceptors (RVEEH HREC study 09-921H, Chief Investigator = Dr Lauren Ayton). The full details of the screening procedures can be found in the BVA SOP PP:C:1, and include visual acuity, fundus photography, fundus autofluorescence (FAF) imaging, optical coherence tomography (OCT), electrophysiology, phosphene perception, visual field assessment, auto-refraction, keratometry, ocular ultrasound, quality of life questionnaires and psychological screening tests and blood samples.

If a patient meets all eligibility criteria at this stage, then the 24 Channel percutaneous plug study will be explained to them verbally and face-to-face (as shown in the timeline in section 4.2). If they are interested in participating, we will go through the PICF verbally and obtain written informed consent. Participants will be informed at this stage that they still might not be eligible for the study, as it will depend on their general

health and psychological status. These factors will be assessed through a general health examination by the surgeon and/or anaesthetist, thorough evaluation of their medical records, and assessment by an independent psychologist.

Aside from the above clinical screening tests, we will require participants to undergo three pre-surgical assessment visits.

1. Psychological assessment: Participants will be asked to see an independent psychologist before the surgery. This is analogous to the procedures followed before organ transplantation surgeries. The first visit will be for assessment of psychological state, including screening for personality disorders, psychiatric illness, anxiety and depression. If the psychologist deems that the person would be psychologically fit and has realistic expectations for the study, then they would offer another visit before surgery for advice and support counselling. Full psychological care will be offered following the surgery, both during and after the study lifespan. The patient will not incur out-of-pocket costs. We would anticipate each subject having one or two sessions prior to device implantation, of approximately 2 hours each. We will also schedule a review appointment with the psychologist prior to the use of a camera in the extended study.

2. General health assessment: Approximately two weeks prior to the surgery the participant will undergo a general health assessment with the surgeon and/or anaesthetist. Medical records from their treating specialists and general practitioner will also be assessed to ensure there are no general health conditions or co-existing morbidities that may exclude them from this study. We would expect the general health assessment to take approximately 2 hours.

3. Orientation and mobility (O&M) assessment: We will obtain information about the participant's orientation and mobility ability prior to surgery. These assessments will be made by assessing the percentage of preferred walking speed[15,16] the patient is able to perform with and without their aids (cane, dog etc.). We will also look at their ability to avoid obstacles. This assessment will be carried out in the hospital environment, the subject's home environment and in an outside environment, and will be videotaped (with patient consent) to allow later analysis. The participant will be monitored at all times by researchers experienced in orientation and mobility research, or a professional certified O&M instructor. If at any time the patient becomes at risk for personal injury, the researchers will intervene verbally (call to "stop") and or physically, as required. Likewise, if they become disoriented they will be given verbal and or physical assistance, as required.

6.2. Trial procedures

6.2.1. Surgical procedures

The surgery for insertion and removal of the 24 channel WPCD will be performed by an experienced team of surgeons at the Royal Victorian Eye and Ear Hospital.

The surgery involves insertion of the electrode array into the suprachoroidal space and positioning of the lead wire beneath the skin to behind the ear, where the percutaneous connector will be sited.

An incision will be made behind the ear and a tunnel formed from this incision to another incision at the corner of the eye. The device and lead wire will be passed through the tunnel and stabilised on the bone at the orbital margin. The conjunctiva will be incised or lifted away from the wall of the eye and a wound made in the sclera behind the lateral rectus muscle. The device will then be inserted into the suprachoroidal space to beneath the macula and the wounds sutured.

The percutaneous connector will stabilised on the skull with screws to the skull and the skin wound sutured around this.

Details of the surgical procedure are included in BVA SOP PP:S:1 and PP:S:2.

Post-surgical care (including medical assessment of the wounds and clinical assessment of retinal stability, retinal appearance etc.) will be closely adhered to. The surgeon will evaluate the wounds and eye daily for the first five days, then on an as-needed basis depending on individual recovery. The length of the hospital stay will be decided on clinical need; estimated to be between 1-5 days. After hospital discharge, the patient will be seen as required according to clinical need by the surgical team for one month. At these visits, retinal stability will also be recorded using an optical coherence tomography (OCT) scan and retinal photos will be taken.

From the second month, if all is progressing well, these medical evaluations are likely to reduce in frequency to around once a month.

Full details of post-operative care and follow up are contained in BVA SOP PP:S:3 and PP:S:4.

It is important to note that after the 2nd month the patient will be coming in to CERA/BI at least once a week for up to 2 years and this will allow close monitoring for any adverse effects. If the clinical staff in the team have any concerns regarding the patient's health (including suspicions of wound infection, implant instability or patient pain) they will contact the surgeon immediately. The patient will also be given a 24 hour emergency mobile phone number, so that they may seek medical attention if required at any time.

6.2.2. Electrical testing protocol

The electrical impedance between pairs of electrodes will be measured automatically as soon as possible after surgery (with very small electrical currents), and then at weekly intervals until the impedance stabilises. Thereafter, the electrical impedance will be measured during psychophysics test sessions as per BVA SOP PP:PT:2. The purpose of these electrical measurements is to detect broken wires and/or short-circuits between electrodes, and to monitor changes in electrode impedance over time to assist in the development of electronics for fully implantable devices.

6.2.3. Psychophysical testing protocol- PHASE 1

During phase 1, we will use the psychophysics system to investigate the characteristics and methods of control of phosphenes generated by electrical stimulation of one electrode at a time in a variety of different electrode configuration modes. A phosphene is an artificial visual percept created when visual nerves are stimulated electrically.

Psychophysical testing will occur in specialised testing suites at the Bionics Institute, Mollison House (on the corner of Lansdowne St and Albert St, one block from the RVEEH).

Full details of the psychophysics testing procedures is found in BVA SOP PP:PT:3 and PP:PT:4

Phase 1 will address the basic question relating to efficacy, and allow the derivation of an initial library of phosphenes (hereafter called a phosphene map), suitable for basic vision processing.

6.2.4. Psychophysical testing protocol - PHASE 2

During Phase 2, participants will be asked to continue attending once a week to perform more detailed and extensive psychophysical testing to ealuate the perception of more complex stimulation patterns including simultaneous stimulation of electrodes and dynamic stimulation patterns. If the participant does not wish to continue into phase 2 for any reason the experiment will stop at this stage.

Full details of the psychophysics testing procedures is found in BVA SOP PP:PT:3 and PP:PT:4

A comprehensive list of research questions has been compiled for investigation using standard psychophysical testing methods. The software and hardware that make up the psychophysics system will be capable of delivering the complex stimulation patterns required to answer the research questions.

The data from Phase 2 psychophysics will provide essential information for the design and optimisation of vision processing strategies.

6.2.5. Psychophysical testing protocol – PHASE 3

In Phase 3, some level of psychophysics testing in the laboratories at BI will continue (i.e. Phase 2), but the focus will be on clinical testing of the patients in more visually challenging environments. Participants will have the option of coming in for one or two days per week.

The data from Phase 3 will be directly applicable to future devices, enabling researchers to develop improved vision processing algorithms and modify the external hardware required for such a setup.

7. Subject Completion and Discontinuation

7.1. Subject completion

If there are any complications with the WPCD, such as pain, infection or danger to the eye or the patient, the entire device will be removed as soon as possible by our surgical team.

However, if the device is stable and not causing any problems, we will leave the electrode array in place until an upgraded, implantable version is available and the ability to safely replace the device has been proven. This is to ensure that the suprachoroidal space remains open, to allow insertion of a replacement device in the future. For safety and cosmetic reasons, the percutaneous connector and wires will be surgically removed at the end of the study, regardless of the decision whether or not to leave the electrode array in place.

7.2. Stopping rules / Discontinuation criteria

If the surgery causes significant trauma to the eye and the implant is not safely inserted, the study will be stopped.

If the stimulation of any electrode causes pain, that electrode will not be used at that level of voltage or greater, at any future time. If it is found that it is not possible to stimulate the retina without pain, the study will be stopped.

7.3. Subject withdrawal

If a participant chooses to have a safe implant removed early, due to cosmetic or personal reasons, counselling on the risks will be provided. If they still wish to proceed with removal, it will be performed at the earliest time available with the surgeon.

7.4. Early termination of the study

The study may be terminated prematurely by the principal investigator or her delegate and the sponsor if:

- The number and/or severity of adverse events justify discontinuation of the study
- New data become available which raise concern about the safety of the device, so that continuation might cause unacceptable risks to subjects.

In addition the Sponsor reserves the right to discontinue the trial prior to inclusion of the intended number of subjects, but intends only to exercise this right for valid scientific or administrative reasons.

After such a decision, the Investigator must contact all participating subjects within two weeks, and written notification must be sent to the Ethics Committee.

8. Case Report Form (CRF)

A Case Report Form (CRF) will be completed for each study subject summarising all clinical screening and study data. Subjects will only be referred to in the CRF by their subject number and initials in order to retain subject confidentiality.

The completed original CRFs are to be sent to the Sponsor as soon as practical after completion and review. A copy of each completed CRF is to be retained by the Investigator for a period of time as determined by local regulations.

The identification of data to be recorded directly in to the CRF (i.e. no prior written or electronic record of data), and to be considered to be source data, is outlined in BVA GCP SOP 24Ch:10

9. Data Analysis and Statistical Considerations

As this pilot study only has a sample size of 3, we are not expecting to be able to complete between-subject statistical analyses. This is an exploratory study to determine the feasibility of suprachoroidal stimulation in humans with retinal degenerative disease, and to collect the psychophysical data to facilitate the development of the future generation bionic eye devices.

The aim of this pilot study will be to collect data on an individual basis, in order to investigate the efficacy of the 24 channel WPCD, including whether the suprachoroidal approach is feasible both surgically and for stimulation requirements.

To determine the efficacy of the device, the level of visual function and functional vision before and after implantation will be compared. The level of visual function and functional vision when the device turned ON will also be compared with that when the device is OFF.

A full record of all eligible subjects will be kept in the screening and enrolment log (section 10.0 of the GCP trial master file).

10. Data Management

Data will be stored in hard copy on the CRFs, and relevant data will be uploaded onto the BVA OpenClinica database or study-specific Excel spreadsheets at the end of each week. The hard copy CRFs will also be scanned and stored electronically at the completion of each Schedule of testing. All electronic databases will be locked with a password, known only to the named investigators of the study.

The specifics of the data collection and testing procedures are outlined in the BVA SOPs, namely PP:C:1 to PP:C:12, PP:S:1 to PP:S:4 and PP:PT:3 and PP:PT:4.

11. Monitoring and Quality Assurance

The independent monitor for this study will be Mobius Medical Pty Ltd.

The task of the Study Monitor is to guarantee the best conduct of the study through contact by phone and in person with the responsible Investigator, in accordance with the Monitor's Standard Operating Procedures, with the purpose of facilitating the work and fulfilling the objectives of the study. These site visits will enable the Monitor to maintain current, personal knowledge of the study through review of the records, comparison with source documents, and observation and discussion of the conduct of the study with the Investigator. The Monitor is responsible for monitoring adherence to the Protocol and completion of the study files, and for the relationship between the Investigator and the Sponsor.

The organisation, monitoring, supply of study materials and quality assurance of the present clinical study is the responsibility of the Sponsor or its designee.

In order to ensure the accuracy of data, direct access to source documents by the representatives of both the Study Monitor and regulatory authorities is mandatory. Anonymity of the subject will be maintained at all times. The Sponsor reserves the right to terminate the study for refusal of the Investigator/Institution to supply source documentation of work performed in the study.

Internal monitoring visits and audits will also be provided by an independent monitor, who works for the Centre for Eye Research Australia but is not associated with this study. This monitor will be responsible for six monthly reviews of the project and all associated documentation.

11.1. Curriculum Vitae and other documentation

In order to comply with regulatory requirements, all Investigators signing the Protocol and all trial staff should provide a current, signed and dated Curriculum Vitae (CV) to be filed in alphabetical order. The CV should include name, title, occupation, education, research experience and present and former positions. A delegation of authority log (9.2 of Trial Master File) contains signatures of all investigators and staff. CVs are included in section 9.3 of the Trial Master File.

12. Investigator Responsibility

Except where the Principal Investigator's signature is specifically required, it is understood that the term 'Investigator' as used in this Protocol and on the CRFs refers to the Principal Investigator or an appropriately qualified member of the staff that the Principal Investigator delegates to perform specified duties of the Protocol. The Principal Investigator is ultimately responsible for the conduct of all aspects of the study.

Each Investigator will comply with the local regulations regarding clinical trials and the Investigator responsibilities outlined in the ICH GCP guidelines.

13. Study Report

A full report on the outcomes of the study will be provided to the Sponsor (CERA) and to BVA at the completion of the study period. Subjects will be provided with verbal updates on their progress during the study period, and will also be provided with a written report at the end of the study.

The report will be prepared by the named investigators on the study, and reviewed/ approved by the principal investigator before submission to the Sponsor.

14. Administrative Procedures

14.1. Ethical considerations

Information on side effects of the test and reference formulations is summarised in the Investigator's Brochure. The amount of blood to be sampled in the study is not considered to be excessive in healthy adult subjects. This study will be carried out according to the Declaration of Helsinki, the NHMRC National Statement on Ethical Conduct in Research Involving Humans (1999) and the Notes for Guidance on Good Clinical Practice as adopted by the Australian Therapeutic Goods Administration (2000) (CPMP/ICH/135/95) and the ICH GCP Guidelines[17].

14.2. Ethical review committee

The Protocol will be submitted for approval to the RVEEH HREC, and written approval obtained. A copy of the respective approval letters will be included in the Trial Master File before starting the study. The composition of the Ethics Committee will also be included in the Trial Master File. If approval is suspended or terminated by the Ethics Committee, the Investigator will notify the Sponsor immediately.

It is the responsibility of the Investigator to report study progress to the Ethics Committee as required or at intervals not greater than one year.

The Principal Investigator, or her delegate, will be responsible for reporting any serious adverse events to the Ethics Committee as soon as possible, and in accordance with the guidelines of the Ethics Committee.

14.3. Regulatory authorities

The Therapeutic Goods Administration (TGA) has been notified of this study using a Clinical Trials Notification (CTN) form, sent 5 April 2012. Notification of CTN processing was received from the TGA on 20 April 2012, and this study has the CTN number 090/2012.

The investigators will abide by the reporting of SAE rules and other regulatory requirements. In agreeing to the provisions of the Protocol, these responsibilities are accepted by the Investigator.

14.4. Informed consent

Before recruitment and enrolment into the study, each prospective candidate will be given a full explanation of the nature and purposes of the study, and a copy of the Subject Information Sheet to review in the individual's preferred format. Once the essential study information has been provided, and the Investigator is assured that each individual volunteer understands the implications of participating in the study, the subjects will be asked to give consent to participate in the study by signing the informed consent form. The consent forms shall be signed and dated by the appropriate parties. Due to the fact that the participants in this study are blind, if they are unable to date their own signature, a witness will do so for them. A notation that written informed consent has been obtained will be made in the subject's study file. The completed consent forms will be retained by the Investigator and a copy of these will be provided by the Investigator to the subject.

14.5. Subject reimbursement

Each subject will be reimbursed for out of pocket expenses, inconvenience and time involved. Such reimbursement is standard practice in studies such as this. If the study is terminated by the Sponsor or the Investigator(s) prior to completion or a subject withdraws or is withdrawn from the study before completion, a pro-rata payment will be made at the discretion of the Investigator(s). Reserve subjects will also be reimbursed for inconvenience and time involved.

14.6. Emergency contact with investigators

All subjects will be provided with a Subject Emergency Contact Card with contact details of whom to contact in the case of an emergency.

14.7. Notification of primary care physician

With the consent of the volunteer, it is the Investigator's responsibility to notify the primary care physician of the subject's participation in the study, provided that such a physician can be identified for the subject. A letter will be sent to the physician stating the nature of the study, treatments, expected benefits or adverse events and concomitant drugs to be avoided. A copy shall be retained by the study site for verification by the Sponsor.

14.8. Investigator indemnification

The study is being conducted subject to the 'Guidelines for Compensation for Injury Resulting from Participation in a Company-sponsored Clinical Trial' published by the Medicines Australia. In the event that the participant suffers an injury as a result of participating in this research project, hospital care and treatment will be provided by the public health service at no cost to them.

14.9. Financial aspects

The conduct of the study, including financial arrangements, is subject to annual Project Agreements between Bionic Vision Australia and the Investigator's institution.

14.10. Protocol amendments

Neither the Investigator nor the Sponsor will modify the Protocol without first obtaining the concurrence of the other in writing. Protocol modifications that impact on subject safety or the validity of the study will be approved by the Ethics Committee.

No changes (amendments) to the Protocol may be implemented without prior approval from the Sponsor and the appropriate Ethics Committee. If a Protocol amendment requires changes to the Informed Consent Form, the revised Informed Consent Form, prepared by the Investigator, must be approved by the Ethics Committee.

Once the final Protocol has been issued and signed by the Investigator and the authorised signatories, it shall not be informally altered. Protocol amendments are alterations to a legal document and have the same legal status. Therefore, they must pass through appropriate steps before being implemented. In general, any important change that theoretically increases risk to subjects constitutes an amendment. Minor changes are administrative changes and need documentation without approval.

It is the responsibility of the Investigator to submit the amendment to the Ethics Committee for their approval; written approval should be obtained and a copy provided to the Sponsor. The Sponsor is responsible for determining whether or not the local regulatory authority must be notified of the Protocol change. Completed and signed Protocol amendments will be circulated to all those who were on the circulation list for the original Protocol.

The original signed copy of amendments will be kept in the Trial Master File with the original Protocol. It should be noted that where an amendment to the Protocol substantially alters the study design or the potential risks to the subjects, each subject's consent to continue participation should be obtained.

14.11. Protocol compliance

The instructions and procedures specified in this Protocol require diligent attention to their execution. Should there be questions or consideration of deviation from the Protocol, clarification will be sought from the Study Monitor. Any subject treated in a manner that deviates from the Protocol, or who is admitted into the study but is not qualified according to the Protocol as amended by the Sponsor and the Investigator, may be ineligible for analysis and thereby compromise the study.

Only when an emergency occurs that requires a departure from the Protocol for an individual will there be such a departure. The nature and reasons for the Protocol violation shall be recorded in the participant study file.

The Investigators and delegates will comply with all applicable federal, state and local laws.

14.12. Archives: Retention of study records

All source documents, CRFs and trial documentation will be kept by the Investigator for the appropriate retention period as stipulated by local regulations and ICH-GCP[17].

15. References

1. Chader GJ, Weiland J, Humayun MS. Artificial vision: needs, functioning, and testing of a retinal electronic prosthesis. *Prog Brain Res* 2009;175:317-32.
2. Bunker CH, Berson EL, Bromley WC, et al. Prevalence of retinitis pigmentosa in Maine. *Am J Ophthalmol* 1984;97(3):357-65.
3. Zrenner E, Bartz-Schmidt KU, Benav H, et al. Subretinal electronic chips allow blind patients to read letters and combine them to words. *Proc Biol Sci* 2011;278(1711):1489-97.
4. Ahuja AK, Dorn JD, Caspi A, et al. Blind subjects implanted with the Argus II retinal prosthesis are able to improve performance in a spatial-motor task. *Br J Ophthalmol* 2010;95(4):539-43.
5. Hood DC, Lin CE, Lazow MA, et al. Thickness of receptor and post-receptor retinal layers in patients with retinitis pigmentosa measured with frequency-domain optical coherence tomography. *Invest Ophthalmol Vis Sci* 2009;50(5):2328-36.
6. Lakhanpal RR, Yanai D, Weiland JD, et al. Advances in the development of visual prostheses. *Curr Opin Ophthalmol* 2003;14(3):122-7.
7. Humayun MS, Dorn JD, Ahuja AK, et al. Preliminary 6 month results from the Argus II epiretinal prosthesis feasibility study. *Conf Proc IEEE Eng Med Biol Soc* 2009:4566-8.
8. Fujikado T, Morimoto T, Kanda H, et al. Evaluation of phosphenes elicited by extraocular stimulation in normals and by suprachoroidal-transretinal stimulation in patients with retinitis pigmentosa. *Graefes Arch Clin Exp Ophthalmol* 2007;245(10):1411-9.
9. Shivdasani MN, Luu CD, Cicione R, et al. Evaluation of stimulus parameters and electrode geometry for an effective suprachoroidal retinal prosthesis. *J Neural Eng* 2010;7(3):036008.
10. Cicione R, Shivdasani MN, Fallon JB, et al. Efficacy of Electrode Return Configuration in a Suprachoroidal Retinal Prosthesis. *Association for Research in Vision and Ophthalmology (ARVO) annual meeting*. Ft. Lauderdale, FL.2011.
11. Nayagam DA, Villalobos J, Allen PJ, et al. A Suprachoroidal Retinal Prosthesis Is Safe In A Chronic Implantation Model. *Association for Research in Vision and Opththalmology (ARVO) annual meeting*. Ft Lauderdale, FL2011.
12. Shivdasani MN, Fallon JB, Luu CD, et al. Cortical Responses to Single and Multiple-Electrode Stimulation of a Suprachoroidal Retinal Prosthesis. *Association for Research in Vision and Ophthalmology (ARVO) annual meeting*. Ft Lauderdale, FL2011.
13. Villalobos J, Nayagam DA, Allen PJ, et al. A Wide-Field Suprachoroidal Retinal Prosthesis Reliably Elicits Cortical Activity after Chronic Implantation. *Association for Research in Vision and Ophthalmology (ARVO) annual meeting*. Ft Lauderdale, FL.2011.
14. Abbas PJ, Brown CJ. Electrically evoked auditory brainstem response: growth of response with current level. *Hear Res* 1991;51(1):123-37.
15. Haymes S, Guest D, Heyes A, Johnston A. Mobility of people with retinitis pigmentosa as a function of vision and psychological variables. *Optom Vis Sci* 1996;73(10):621-37.
16. Haymes S, Guest D, Heyes A, Johnston A. Comparison of functional mobility performance with clinical vision measures in simulated retinitis pigmentosa. *Optom Vis Sci* 1994;71(7):442-53.
17. Note for Guidance on Good Clinical Practice (CPMP/GCP/135/95) and Note for Guidance on Good Clinical Practice (CPMP/GCP/135/95) annotated with Therapeutic Goods Administration (TGA) comments (DSEB, July 2000)
